# In Vitro Selection of Macrocyclic l-α/d-α/β/γ-Hybrid Peptides Targeting IFN-γ/IFNGR1 Protein–Protein Interaction

**DOI:** 10.1021/jacs.4c01979

**Published:** 2024-06-18

**Authors:** Takashi Miura, Kang Ju Lee, Takayuki Katoh, Hiroaki Suga

**Affiliations:** Department of Chemistry, Graduate School of Science, The University of Tokyo, 7-3-1 Hongo, Bunkyo-ku, Tokyo 113-0033, Japan

## Abstract

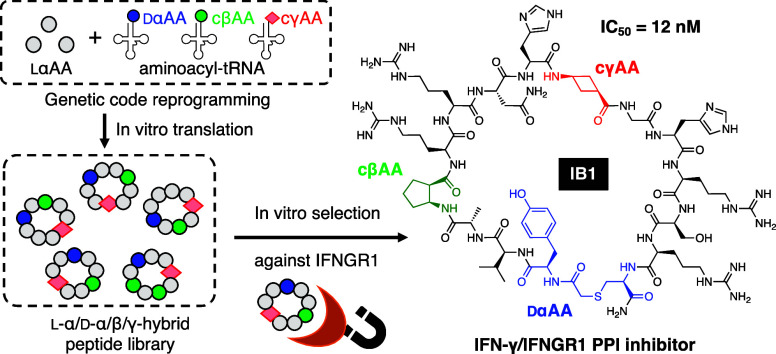

Nonproteinogenic amino acids, including d-α-, β-, and γ-amino acids, present in bioactive peptides play pivotal roles in their biochemical activities and proteolytic stabilities. d-α-Amino acids (dαAA) are widely used building blocks that can enhance the proteolytic stability. Cyclic β^2,3^-amino acids (cβAA), for instance, can fold peptides into rigid secondary structures, improving the binding affinity and proteolytic stability. Cyclic γ^2,4^-amino acids (cγAA) are recently highlighted as rigid residues capable of preventing the proteolysis of flanking residues. Simultaneous incorporation of all dαAA, cβAA, and cγAA into a peptide is expected to yield l-α/d-α/β/γ-hybrid peptides with improved stability and potency. Despite challenges in the ribosomal incorporation of multiple nonproteinogenic amino acids, our engineered tRNA^Pro1E2^ successfully reaches such a difficulty. Here, we report the ribosomal synthesis of macrocyclic l-α/d-α/β/γ-hybrid peptide libraries and their application to in vitro selection against interferon gamma receptor 1 (IFNGR1). One of the resulting l-α/d-α/β/γ-hybrid peptides, IB1, exhibited remarkable inhibitory activity against the IFN-γ/IFNGR1 protein–protein interaction (PPI) (IC_50_ = 12 nM), primarily attributed to the presence of a cβAA in the sequence. Additionally, cγAAs and dαAAs in the resulting peptides contributed to their serum stability. Furthermore, our peptides effectively inhibit IFN-γ/IFNGR1 PPI at the cellular level (best IC_50_ = 0.75 μM). Altogether, our platform expands the chemical space available for exploring peptides with high activity and stability, thereby enhancing their potential for drug discovery.

## Introduction

**Figure 1:**
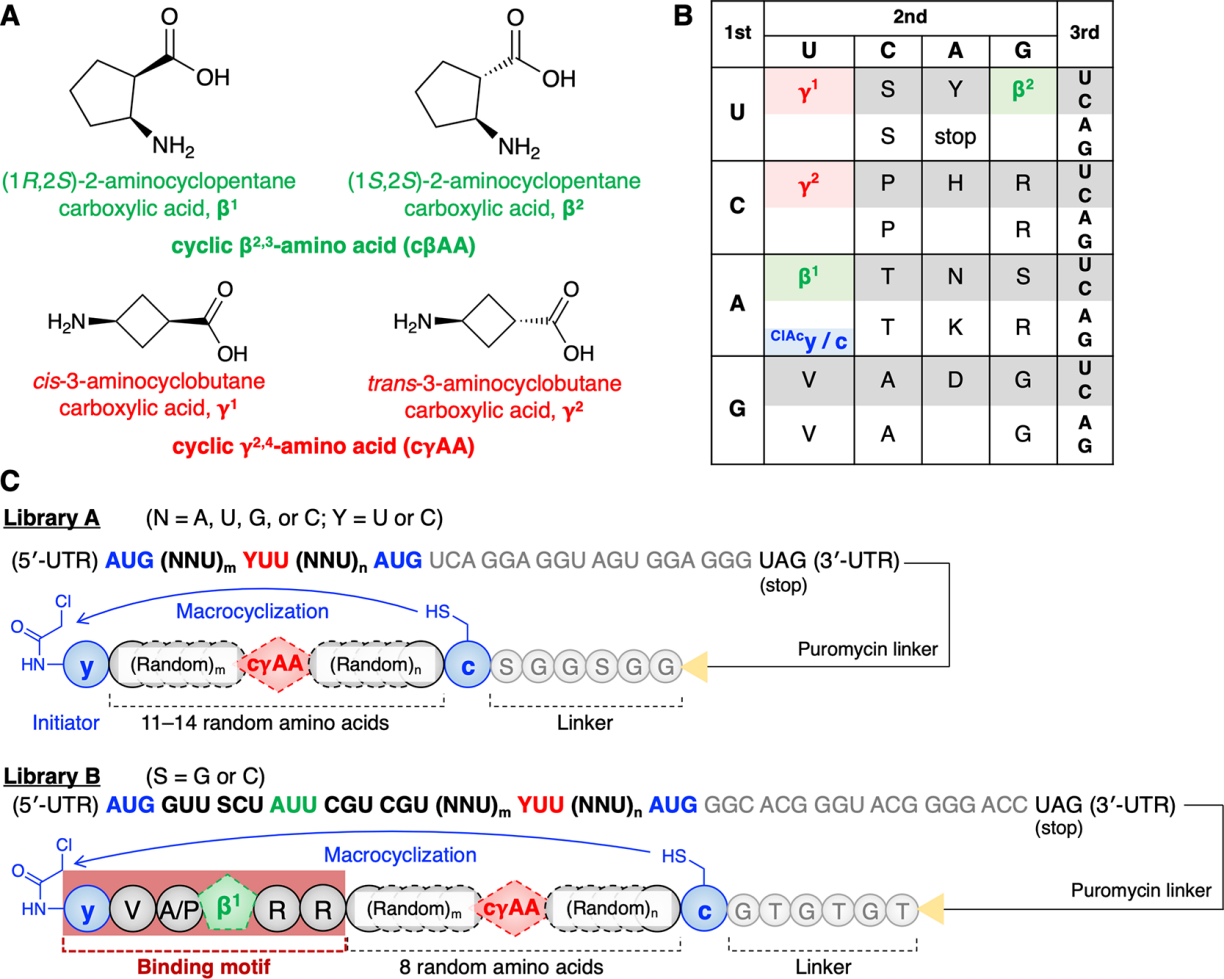
Design of macrocyclic l-α/d-α/β/γ-hybrid peptide libraries. (A) Structures of cβAAs and cγAAs used in this study. (B) Reprogrammed codon table that consists of two dαAAs (^ClAc^y and c), two cβAAs (β^1^ and β^2^), two cγAAs (γ^1^ and γ^2^), and proteogenic amino acids. ^ClAc^y was assigned at the initiator AUG codon, while the other amino acids were assigned at elongator codons. (C) Sequence design of the random mRNA libraries and the corresponding peptide libraries. Peptides spontaneously macrocyclized between ^ClAc^y and c via a thioether bond. The mRNA and peptide were covalently linked via a puromycin linker after translation.

Bioactive peptides reported to date consist not only of proteinogenic l-α-amino acids and achiral glycine but also of diverse nonproteinogenic amino acids, such as d-α-, β-, and γ-amino acids, as well as macrocyclic scaffolds.^1−6^ Such nonstandard macrocyclic peptides are considered promising drug candidates due to their higher binding affinity to targets, proteolytic resistance, and membrane permeability compared to peptides consisting solely of proteinogenic amino acids, owing to the contributions of various nonproteinogenic amino acids.^6^d-α-Amino acids (dαAA) are known to improve proteolytic resistance of peptides against various endogenous proteases.^7−10^ β-Amino acids can enhance the structural rigidity of peptides by inducing unique helical and turn structures, which are generally more stable than the α-helix and β-turn structures in α-peptides.^11−15^ Among them, cyclic β^2,3^-amino acids (cβAA; refer to Figure 1A for the structures of representative cβAAs used in this study), bearing constrained cyclic main chains, can fold peptides into particularly rigid secondary structures, such as 12-helix, 14-helix, 10/11/11-helix, β-turn, and γ-turn.^16−22^ This distinctive folding propensity of β-peptides can enhance the binding affinity to target proteins by minimizing the entropic penalty and improving proteolytic resistance against various proteases.^23,24^ γ-Amino acids represent another class of appealing building blocks capable of inducing rigid helix and turn structures, such as 14-helix and 12-turn, and improving proteolytic stability of peptides.^23,25−28^ In particular, 3-aminocyclobutane carboxylic acids, characterized by four-membered rings (see Figure 1A for their structures), are compact and rigid cyclic γ^2,4^-amino acids (cγAA) that can significantly enhance proteolytic stability of given peptides when substituted for small amino acids, such as Ala, as recently reported by our group.^29^

Taking advantage of the remarkable properties of these nonproteinogenic amino acids, high-throughput screening of nonstandard peptides from ribosomally synthesized libraries, utilizing genetic code reprogramming, has been conducted for discovering novel bioactive peptides.^30−32^ To ribosomally synthesize nonstandard peptides, we have developed the flexible in vitro translation (FIT) system which comprises the reconstituted*Escherichia coli*translation system with precharged nonproteinogenic aminoacyl-tRNA prepared by the flexizymes.^33,34^ The FIT system allows the construction of random peptide libraries, comprising over 10^12^ unique members, applicable to an in vitro selection methodology, referred to as the Random nonstandard Peptides Integrated Discovery (RaPID) system, based on the mRNA display method.^30,35^ We constructed macrocyclic peptide libraries containing combinations of l-α-, d-α-, β-, and γ-amino acids, such as [l-α- and d-α-amino acids],^10^ [l-α- and β-amino acids],^36^ and [l-α-, d-α-, and β-amino acids],^24,36−38^ which were then applied to the RaPID system against various targets. The selection results revealed the contribution of d-α- and β-amino acids to the proteolytic stability of peptide inhibitors and the crucial role of cβAA residues in inhibitory activity by folding peptides into rigid β-sheets. Very recently, we reported the first in vitro selection of macrocyclic peptide inhibitors consisting of l-α-amino acids, dαAAs, and cγAAs against the SARS-CoV-2 main protease.^29^ Each selected peptide contained one cγAA, which conferred resistance to degradation by the main protease and the serum peptidases.

Given the contributions of d-α-, β-, and γ-amino acids to proteolytic stability and inhibitory activity of peptides, the incorporation of all l-α-, d-α-, β-, and γ-amino acids into a single peptide sequence at once, i.e., l-α/d-α/β/γ-hybrid peptides, is expected to yield a bioactive peptide with improved proteolytic stability and high potency against various therapeutic targets. A library of l-α/d-α/β/γ-hybrid peptides, having distinct backbone structures, can expand the chemical space accessible through screening, thereby increasing the possibility of selecting peptides that exhibit stronger binding to the target. On the other hand, the construction of a library of l-α/d-α/β/γ-hybrid peptides is yet challenging due to their intrinsic poor efficiency of incorporation into a nascent peptide backbone chain; moreover, the downstream selection using such a library has not been accomplished due to serious difficulties in the multiple and/or consecutive incorporation of d-α-, β-, and γ-amino acids.^39−43^ The low efficiencies are attributed to the slow accommodation of nonproteinogenic aminoacyl-tRNA onto the ribosome and sluggish peptidyl transfer reaction between the nonproteinogenic amino acid and its flanking residues.^44,45^ To address these challenges, we have developed a chimeric body sequence based on tRNA^Glu^ T-stem and tRNA^Pro1^ D-arm, referred to as tRNA^Pro1E2^ (Supporting Information (SI) Figure S1A); this tRNA is able to effectively recruit EF-Tu and EF-P to improve accommodation to the ribosome A site and to avoid rapid peptidyl-tRNA drop-off from the ribosome P site, respectively.^46,47^ As a result, tRNA^Pro1E2^ charged with d-α-, β-, and γ-amino acids enables their incorporation into a nascent peptide chain with significantly improved efficiencies,^24,48−53^ making achievable for the construction of macrocyclic peptide libraries with a combination of l-α-, d-α-, and β-amino acids, or l-α-, d-α-, and γ-amino acids. This has led us to discover potent binders and inhibitors of macrocycles containing such a combination of exotic amino acids against proteins of interest.^10,24,29,36,37^ On the other hand, a challenge for the construction of a library containing a combination of all of such exotic amino acids and discovery of de novo l-α/d-α/β/γ-hybrid peptides remains unachieved. Here, we report, for the first time, the in vitro selection of macrocyclic l-α/d-α/β/γ-hybrid peptide inhibitors against a therapeutic target, human interferon gamma receptor 1 (IFNGR1).

## Results

### RaPID Selection of Macrocyclic l-α/d-α/β/γ-Hybrid Peptides from a Random Library

To construct a library of macrocyclic l-α/d-α/β/γ-hybrid peptides, we introduced two cβAAs, (1*R*,2*S*)-2-aminocyclopentane carboxylic acid (β^1^) and (1*S*,2*S*)-2-aminocyclopentane carboxylic acid (β^2^), as well as two cγAAs, *cis*-3-aminocyclobutane carboxylic acid (γ^1^) and *trans*-3-aminocyclobutane carboxylic acid (γ^2^), at the AUU, UGU, UUU, and CUU codons using precharged β^1^-tRNA^Pro1E2^_GAU_, β^2^-tRNA^GluE2^_GCA_, γ^1^-tRNA^Pro1E2^_GAA_, and γ^2^-tRNA^Pro1E2^_GAG_, respectively (library A; Figure 1 and SI Figure S1). For macrocyclization of the peptide, *N*-chloroacetyl-d-tyrosine (^ClAc^y) and d-cysteine (c) were introduced at the initiator and elongator AUG codons using precharged ^ClAc^y-tRNA^fMet^_CAU_ and c-tRNA^Pro1E2^_CAU_, respectively. The thiol group of the c residue spontaneously reacts with the N-terminal chloroacetyl group of ^ClAc^y to form a thioether bond. Each nonproteinogenic amino acid was precharged onto the respective tRNA using flexizymes. Translation was initiated with ^ClAc^y, followed by a repeat of 11–14 random residues encoded by the NNU codons (N = A, U, G, or C), c residue, and an SGGSGG linker connected to the 3′ end of the mRNA via a puromycin linker (Figure 1C). The two cβAAs and the 11 proteinogenic l-α-amino acids (A, D, G, H, N, P, R, S, T, V, and Y) were assigned to the NNU codons. Each peptide was designed to have at least one cγAA encoded by the YUU codons (Y = U or C), strategically placed in the middle of randomized sequence, i.e., encoded by (NNU)_*m*_-YUU-(NNU)_*n*_ (*m*, *n* = 0–13). It should be noted that in the previously reported library (library P; SI Figure S2), both cβAAs and cγAAs were incorporated, but cβAA-containing peptides were not enriched in the major family after selection.^29^ To obtain l-α/d-α/β/γ-hybrid peptides from library A, the number of cβAAs and cγAAs assigned to the 16 NNU codons has increased from 2 to 4. This modification substantially enhanced the possibility of enriching the l-α/d-α/β/γ-hybrid peptides.

Library A was then applied to the RaPID selection against IFNGR1, a receptor of IFN-γ known as a proinflammatory cytokine.^54,55^ An inhibitor of the IFN-γ and IFNGR1 protein–protein interaction (PPI) would be a therapeutic agent for autoimmune diseases. The random mRNA library was translated into the peptide library, followed by conjugation of the peptide with the parent mRNA via a puromycin linker (SI Figure S3A). The mRNA/peptide conjugates were reverse transcribed into mRNA/cDNA/peptide complexes and applied to affinity selection. The library was first subjected to naked magnetic beads to remove bead-binders and then applied to Fc-tagged IFNGR1 immobilized on Protein G magnetic beads to recover IFNGR1-binders. The bound fractions were recovered and amplified into the cDNA library by PCR, followed by transcription into the mRNA library for the next selection round. By repeating this affinity selection, the recovery rate of IFNGR1-binders significantly increased at the fifth round, while those of bead-binders did not increase (SI Figure S3B). The cDNA sequences and the corresponding peptides at the fifth round were analyzed by next-generation sequencing (SI Table S1 shows the top 100 sequences). 58% of the peptides shared a β^1^-containing six-residue motif [yV(A or P)β^1^RR] at their N-termini, whereas cγAA was found in less than 2% of sequences. The peptides without cγAA emerged through mutations during PCR amplification of the cDNA library, and they are considered to have become the majority in the repeated selection rounds due to their higher translation efficiency compared to cγAA-containing peptides. We chose two l-α/d-α/β/γ-hybrid peptides with the conserved motif, IA1 containing γ^1^ and IA2 containing γ^2^ (Table 1). IA1 and IA2 were chemically synthesized on a large scale using the standard solid phase method without the C-terminal SGGSGG linker, and their identities were confirmed by MALDI-TOF MS (SI Figures S4A and S5A).

**Table 1:**
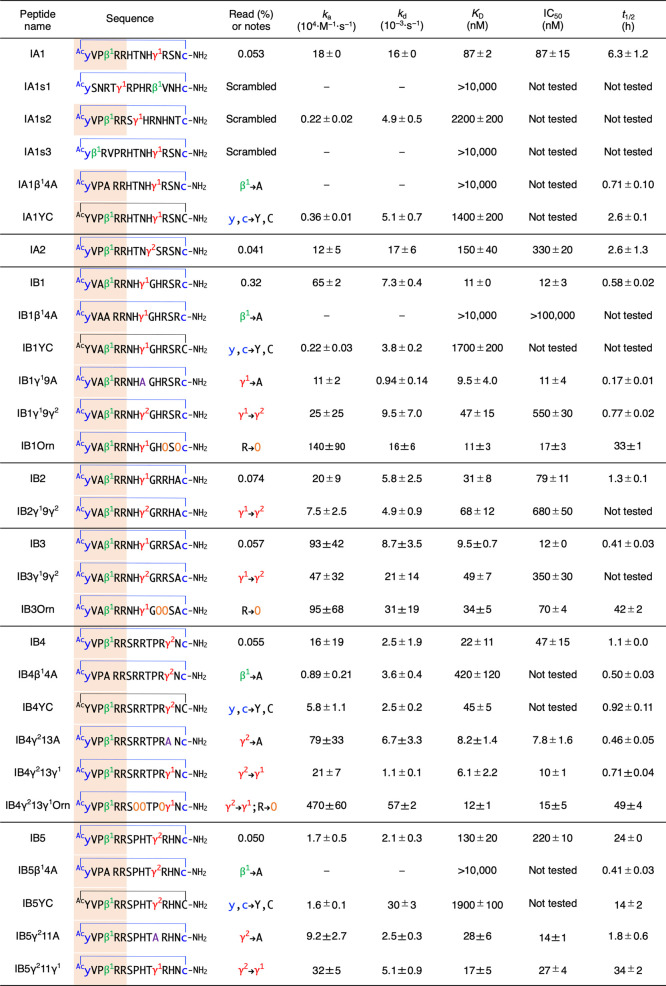
Binding Affinity, Inhibitory Activity, and Serum Stability of Selected Peptides and their Variants^a^

a The thioether bonds are indicated by blue lines where the sulfide atom is omitted. C-terminus of the peptide is amidated. N-terminal [yV(A or P)β^1^RR] binding motif is highlighted in orange. See SI Figure S4 for the structures of peptides. Sequence, read (%), kinetic association (*k*_a_), dissociation (*k*_d_), equilibrium (*K*_D_), half-maximal inhibitory concentration (IC_50_), and half-life in human serum (*t*_1/2_) are shown. *k*_a_, *k*_d_, and *K*_D_ were measured by SPR. IC_50_ for the inhibition of IFN-γ/IFNGR1 interaction was determined by AlphaLISA. *t*_1/2_ was estimated by LC/MS. See Figures 2, 3, S6–S8, and Methods for the details of experiments. See Figure 5 for the cellular inhibitory activity of IB1Orn, IB4γ^2^13γ^1^Orn, and IB5γ^2^11γ^1^ (IC_50_ = 3.5 μM, 0.75 μM, and 1.4 μM, respectively). −: The kinetic values could not be accurately determined due to the low affinity.

Binding affinities of IA1 and IA2 against IFNGR1 were evaluated by surface plasmon resonance (SPR) (Table 1 and SI Figure S6A). *K*_D_ values for IA1 and IA2 were 87 and 150 nM, respectively, while their read frequencies were approximately 0.05%. To investigate the contribution of the N-terminal [yVPβ^1^RR] motif and β^1^ residue in IA1, three scrambled peptides IA1s1–3 and a variant IA1β^1^4A were synthesized and evaluated. As expected, IA1s1, which scrambled the randomized amino acid region between cyclizing ^ClAc^y and c, lost binding affinity to IFNGR1. IA1s2, which retained the N-terminal [yVPβ^1^RR] motif and scrambled the remaining residues, exhibited a *K*_D_ of 2200 nM, while IA1s3, which scrambled the [yVPβ^1^RR] motif and retained the remaining residues, demonstrated no binding, indicating the essential role of the N-terminal motif in binding. Substitution of β^1^ with Ala in the motif led to a loss of binding affinity, likely due to the absence of a turn-inducing amino acid, resulting in a conformational change of the peptide (IA1β^1^4A; Table 1). We next evaluated inhibitory activities of IA1 and IA2 against the ligand–receptor PPI between IFN-γ and IFNGR1 using AlphaLISA (Table 1 and SI Figure S7).^37^ IA1 and IA2 exhibited IC_50_ values of 87 and 330 nM, respectively, consistent with their *K*_D_ values. We also determined the serum half-lives of IA1 and IA2, considering peptidase resistance as an important factor for therapeutic peptides.^24^ A sample peptide and a peptidase-resistant internal standard peptide were coincubated in human serum at 37 °C, and the relative amount of the remaining sample peptide was estimated by LC/MS. Both IA1 and IA2 showed moderate peptidase resistance with half-lives of 6.3 and 2.6 h, respectively (Table 1 and SI Figure S8).

### Subsequent Selection of Macrocyclic Peptides with the Binding Motif

To obtain more potent inhibitors with increased activity and extended serum half-life compared to IA1 and IA2, we constructed a focused library where the N-terminal region was designed to have a fixed [yV(A or P)β^1^RR] binding motif (library B; Figure 1) and the remaining amino acids were randomized. Library B shared the same amino acid set and reprogrammed codon table as those of library A. The peptide library was designed to be initiated with the ^ClAc^yV(A or P)β^1^RR motif encoded by AUG-GUU-SCU-AUU-CGU-CGU (S = G or C), followed by a repeat of eight random amino acids in NNU codons, c residue, and a GTGTGT linker sequence. γ^1^ and γ^2^ assigned at the YUU codons were fixed in the middle of the random sequence. Library B was screened against IFNGR1, and the recovery of IFNGR1-binders significantly increased at the third round, which is two rounds earlier than the initial selection using library A (SI Figure S3C). This was attributed to the conserved binding motif shared by each peptide, allowing shorter rounds of selection. Next-generation sequencing data of the cDNA library at the fourth round showed that over 99% of peptides had the binding motif as we expected, and both cβAA and cγAA were found in more than 90% of sequences (SI Table S1 shows the top 100 sequences). We chose five l-α/d-α/β/γ-hybrid peptides, IB1–5, for large-scale chemical synthesis and subsequent analysis (Table 1, SI Figures S4B and S5B,C).

The binding affinities of IB1–5 with IFNGR1 were evaluated by SPR, revealing that IB1–3 containing γ^1^ and IB4 containing γ^2^ exhibited substantially higher affinities (*K*_D_ = 9.5–31 nM), whereas IB5 containing γ^2^ showed a comparable affinity to IA2 (*K*_D_ of IB5 = 130 nM) (Table 1 and SI Figure S6B,C). In order to confirm the importance of β^1^ and dαAA (cyclizing y and c) residues to affinity, we chemically synthesized variants in which Ala or the corresponding l-amino acids (Y and C) were substituted for β^1^ and dαAAs, respectively, and analyzed their binding affinities (IB1β^1^4A, IB1YC, IB4β^1^4A, IB4YC, IB5β^1^4A, and IB5YC; Table 1, SI Figures S5B,C and S6B,C). The binding affinity of variants, except for IB4YC, dramatically dropped by over 10-fold (2-fold decrease for IB4YC), confirming the pivotal role of β^1^ and dαAAs. We next investigated the contribution of cγAA to the sequence. Variants containing Ala or *cis*/*trans*-isomer (γ^1^ → γ^2^ or γ^2^ → γ^1^) were synthesized, and their *K*_D_ values were evaluated (Table 1). All three Ala variants (IB1γ^1^9A, IB4γ^2^13A, and IB5γ^2^11A) exhibited *K*_D_ values comparable to their originals, which is consistent with our previous report showing that the conformation of cγAA in cyclic peptides is similar to that of small amino acids, including Ala. To our surprise, substitution of γ^2^ for γ^1^ increased the *K*_D_ value (IB1γ^1^9γ^2^, IB2γ^1^9γ^2^, and IB3γ^1^9γ^2^), whereas substitution of γ^1^ for γ^2^ decreased the *K*_D_ value (IB4γ^2^13γ^1^ and IB5γ^2^11γ^1^), indicating the preference for the *cis*-form cγAA (γ^1^) over the *trans*-form (γ^2^) in cyclic peptides, regardless of the position, at least in this case.

Subsequently, inhibitory activities of the potent cyclic peptides, IB1–5, and their variants were analyzed (Table 1, Figure 2, and SI Figure S7). IB1 and IB3 exhibited strong inhibitory activity, both with IC_50_ values of 12 nM, correlating with their strong binding affinities in the SPR analysis (*K*_D_s of 11 and 9.5 nM, respectively). IB2, IB4, and IB5 also showed substantial activity with two- or three-digit nM IC_50_ values. In contrast, IC_50_ of IB1β^1^4A (*K*_D_ > 10,000 nM) was not determined below 100,000 nM, confirming the critical role of β^1^ in the inhibitory activity. Variants in which cγAA was substituted with Ala exhibited activities comparable to or higher than those of the originals (IB1γ^1^9A, IB4γ^2^13A, and IB5γ^2^11A). A comparison of the *cis*/*trans*-isomers of cγAA revealed that peptides with γ^1^ (*cis*-form; IB1, IB2, IB3, IB4γ^2^13γ^1^, and IB5γ^2^11γ^1^) showed 5–46-fold higher activity than those with γ^2^ (*trans*-form).

**Figure 2:**
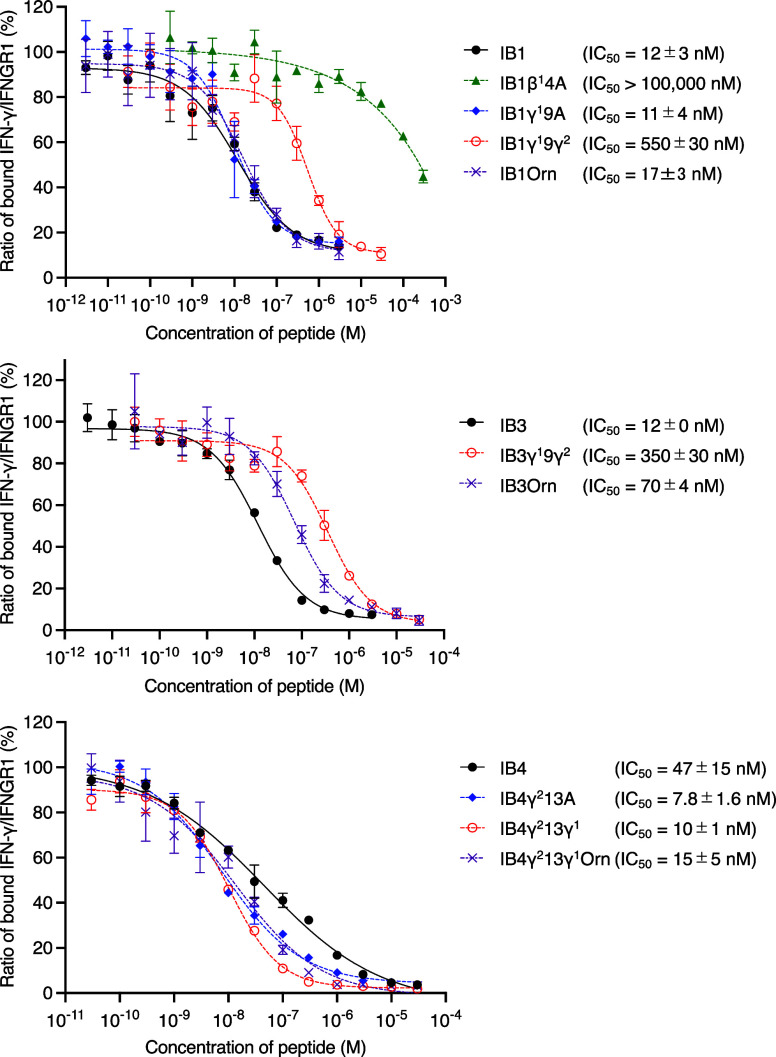
Inhibitory activity of IB1, IB3, IB4, and their variants against IFN-γ/IFNGR1 PPI determined by AlphaLISA. Data are presented as mean values ± standard deviation, SD (*n* = 3). IC_50_ was estimated by a four-parameter dose–response curve fitting using GraphPad Prism 9. See SI Figure S7 for other peptides.

### Serum Stability Assay and Improvement of Half-Life by Ornithine Substitution

**Figure 3:**
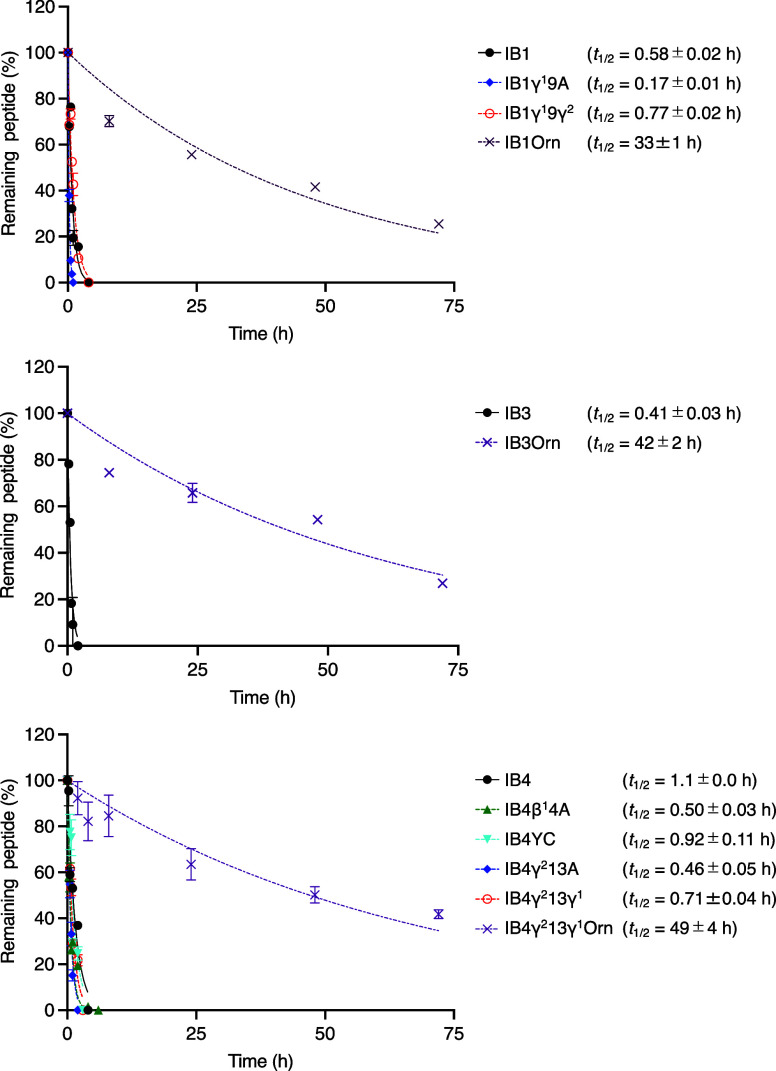
Serum stability assay of IB1, IB3, IB4, and their variants. Each peptide was coincubated with an internal standard peptide in human serum at 37 °C. At each time point, the relative intensity of each peptide to the standard peptide was estimated by LC/MS. Half-life of peptide (*t*_1/2_) was analyzed by nonlinear regression curve fitting using GraphPad Prism 9. Data are presented as mean ± SD (*n* = 3). See the SI Figure S8 for other peptides.

Next, we determined the serum half-life of IB1–5 and their variants (Table 1, Figure 3, and SI Figure S8). IB1–4 exhibited low serum half-lives (*t*_1/2_ = 0.41–1.3 h), while IB5 showed substantially high stability with the half-life of 24 h. Ala variants (IB1γ^1^9A, IB4β^1^4A, IB4γ^2^13A, IB5β^1^4A, and IB5γ^2^11A) and d- to l-amino acid variants (IB4YC and IB5YC) showed half-lives shorter than those of the originals, highlighting the contribution of cβAA, cγAA, and dαAAs to serum stability. Notably, IB5γ^2^11γ^1^ exhibited the longest half-life (*t*_1/2_ = 34 h), although the correlation between the conformation of cγAA and the serum half-life remains unclear. We speculated that the number of Arg residues in a peptide affects its stability, as evidenced by IA1, IA2, and IB5, which have three Arg residues and half-lives of which were over 2.6 h, in contrast to IB1, IB2, IB3, and IB4, which have four or five Arg residues and degraded more rapidly (*t*_1/2_ < 1.3 h).

To identify peptidase cleavage sites in serum, the products of IB1, IB3, IB4, IB4γ^2^13γ^1^, IB5, and IB5γ^2^11γ^1^ at time points near their half-lives were analyzed by LC/MS (IB1-f1–5, IB3-f1–6, IB4-f1–8, IB4γ^2^13γ^1^-f1–4, IB5-f1–4, and IB5γ^2^11γ^1^-f1–4, SI Figure S8B–D). The sequences of IB1-f1, IB3-f1, and IB4-f1 were determined by LC/MS/MS (SI Figure S8U). Since peptides consisting only of proteinogenic amino acids are rapidly degraded by peptidases, only relatively stable fragments are detectable. Most fragments contained the c-(thioether)-yV(A or P)β^1^RR binding motif, indicating that the nonstandard dαAAs (c and y) and cβAA (β^1^) strongly stabilize the binding motif against proteolysis. Additionally, β^1^ was considered to form a stable turn structure and thus protected the Arg residues at the fifth and sixth positions from peptidases. IB1-f1, IB3-f1, IB3-f2, IB4-f1, and IB4γ^2^13γ^1^-f1 suggested that an Arg (R) residue furthest from cβAA and cγAA residues was cleaved first by endopeptidases including trypsin-like proteases (R14 for IB1, R12 for IB3, and R9 for IB4 and IB4γ^2^13γ^1^), resulting in the short half-lives of IB1, IB3, IB4, and IB4γ^2^13γ^1^ (0.58, 0.41, 1.1, and 0.71 h, respectively). In contrast, the R12 residues in IB5 and IB5γ^2^11γ^1^, which showed significantly longer half-lives (24 and 34 h, respectively), were protected by the flanking cγAA residue at the 11th position, effectively preventing proteolysis as previously reported.^29^

**Figure 4:**
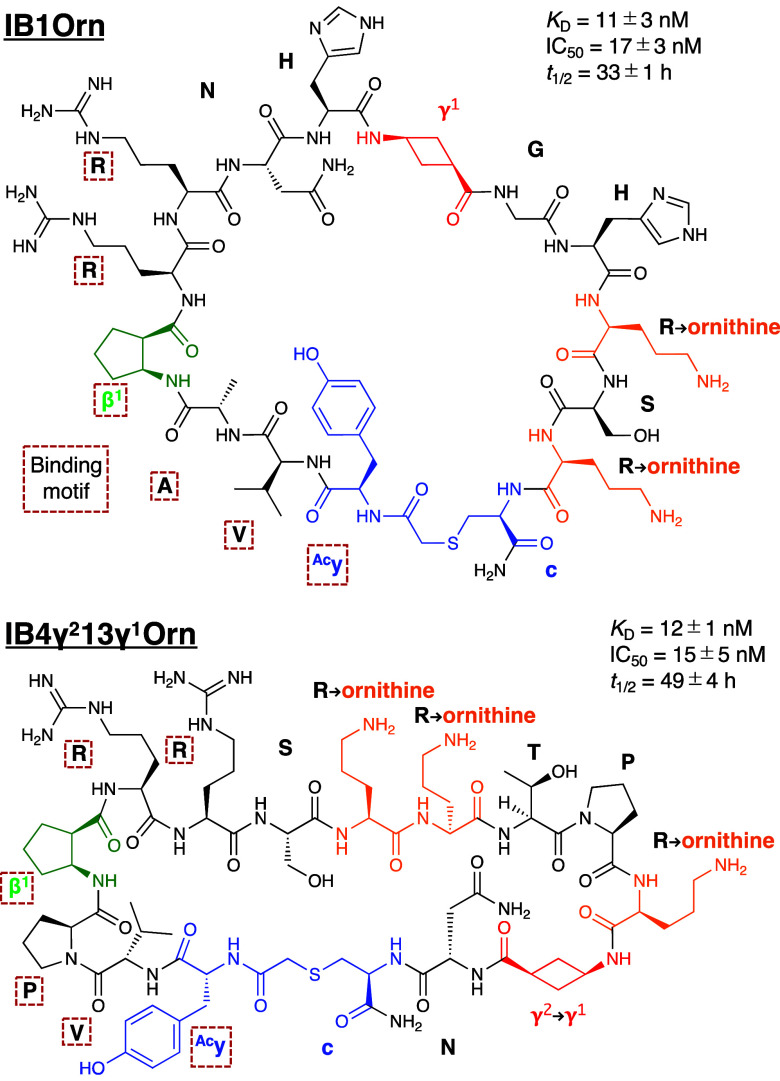
Structures of IB1Orn and IB4γ^2^13γ^1^Orn, which exhibit high binding affinity, inhibitory activity, and serum stability. dαAAs, cβAA, cγAA, and ornithine residues are colored in blue, green, red, and orange, respectively. The labels of binding motif residues are enclosed in red boxes.

To improve the serum stability of potent inhibitors, IB1, IB3, and IB4γ^2^13γ^1^, we synthesized their variants, IB1Orn, IB3Orn, and IB4γ^2^13γ^1^Orn, respectively, where Arg residues not involved in the binding motif were substituted with noncanonical ornithine, known to resist recognition by trypsin-like proteases (Figure 4).^56,57^ As expected, IB1Orn (*t*_1/2_ = 33 h), IB3Orn (*t*_1/2_ = 42 h), and IB4γ^2^13γ^1^Orn (*t*_1/2_ = 49 h) exhibited a significant increase in serum half-life by 57-fold, 100-fold, and 130-fold, respectively, while maintaining inhibitory activities (IC_50_ of IB1Orn = 17 nM, IB3Orn = 70 nM, and IB4γ^2^13γ^1^Orn = 15 nM). Fragment analysis confirmed that the ornithine substitutions (R12O and R14O for IB1Orn; R11O and R12O for IB3Orn; and R8O, R9O, and R12O for IB4γ^2^13γ^1^Orn) stabilized the flanking peptide bonds, while maintaining the stability of the c-(thioether)-yV(A or P)β^1^RR motif (IB1Orn-f1–11, IB3Orn-f1–12, and IB4γ^2^13γ^1^Orn-f1–10, SI Figure S8B,C).

### Cellular Inhibitory Activity Evaluation with HEK-Dual IFN-γ/IFNGR1 Reporter Assay

**Figure 5:**
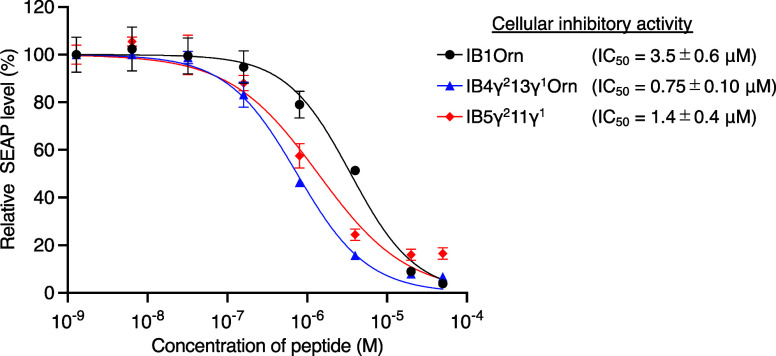
Cellular inhibitory activity of IB1Orn, IB4γ^2^13γ^1^Orn, and IB5γ^2^11γ^1^against IFN-γ/IFNGR1 PPI determined by the HEK-Dual reporter assay. Data are presented as mean ± SD (*n* = 3). IC_50_ value was estimated by a normalized dose–response curve fitting using GraphPad Prism 9.

Lastly, we investigated whether the potent in vitro properties of the peptides could translate into cellular activity. IB1Orn, IB4γ^2^13γ^1^Orn, and IB5γ^2^11γ^1^ were selected for the cell-based assay considering their pronounced in vitro inhibitory activities and prolonged serum stabilities. HEK-Dual reporter cells were used for this purpose, which produce the secreted embryonic alkaline phosphatase (SEAP) into the supernatant in response to the IFN-γ/IFNGR PPI. By quantifying the SEAP amount upon peptide treatment and subsequent IFN-γ stimulation, the inhibitory activity of peptides against IFN-γ/IFNGR PPI can be evaluated at a cellular level. All tested peptides, IB1Orn, IB4γ^2^13γ^1^Orn, and IB5γ^2^11γ^1^, exhibited significant cellular inhibitory activity with IC_50_ values of 3.5, 0.75, and 1.4 μM, respectively, demonstrating that they effectively blocked the IFN-γ/IFNGR PPI at the cellular context (Figure 5). In addition, we assessed the cytotoxicity of the peptides on the same cell line. As expected, none of them exhibited notable cytotoxicity up to 50 μM for 24 h of incubation, demonstrating that the inhibitory signal observed in the reporter gene assay was not derived from general toxicity (SI Figure S9).

## Discussion

This study demonstrated the first in vitro selection of macrocyclic l-α/d-α/β/γ-hybrid peptide inhibitors from highly diverse peptide libraries containing over 10^12^ unique members. We incorporated two dαAAs, two cβAAs, and two cγAAs into the peptide libraries A and B, and each nonproteinogenic amino acid contributed to the binding affinity, inhibitory activity, and serum stability of the selected peptides. Cyclizing dαAAs, ^ClAc^y and c, contributed to the binding affinity of peptides and enhanced their serum half-lives by 1.2–2.4-fold based on the results of IA1YC, IB4YC, and IB5YC, with the substitutions of corresponding l-amino acids, compared to their originals. cβAA greatly contributed to the biochemical activities and stabilities. The β^1^ residue, found in the [yV(A or P)β^1^RR] binding motif, is crucial for binding and inhibitory activity of peptides, as the Ala variants, IA1β^1^4A, IB1β^1^4A, IB4β^1^4A, and IB5β^1^4A, significantly diminished or lost their activities, probably due to the strong folding ability of β^1^.^16^ Moreover, β^1^ enhanced the serum half-lives by 2.2–59-fold and prevented hydrolysis of flanking residues revealed by the fragment analyses (SI Figure S8B–D). cγAAs contributed to the longer serum half-lives of IB1, IB4, and IB5 compared to their Ala variants by 2.4–13-fold. By combining the contributions of dαAAs, cβAAs, and cγAAs, we successfully obtained highly active macrocyclic peptides, such as IB1 and IB4γ^2^13γ^1^, with IC_50_ values of 12 and 10 nM, respectively.

On the other hand, the serum half-lives of IB1 and IB4γ^2^13γ^1^ were rather short (0.58 and 0.71 h, respectively), attributing to the four and five Arg residues present in these sequences. To overcome this limitation, we designed variants, including IB1Orn and IB4γ^2^13γ^1^B4Orn, by substituting the nonessential Arg residues with ornithine, while preserving two essential Arg residues in the [yV(A or P)β^1^RR] binding motif. Notably, IB1Orn and IB4γ^2^13γ^1^B4Orn exhibited substantially prolonged serum half-lives of 33 and 49 h, respectively, while retaining their inhibitory activities (IC_50_ = 17 and 15 nM, respectively). These IFN-γ/IFNGR1 PPI inhibitors demonstrate a balance of activity and proteolytic stability at a high level, compared to the previously reported peptide binders, such as ArβI-3 (IC_50_ = 12 nM, *t*_1/2_ = 11 h)^37^ and I1–5 (*K*_D_ = 1.87 nM, *t*_1/2_ = 66 h, IC_50_ not reported),^24^ both containing two cβAAs and cyclizing ^ClAc^y and c from different libraries. Notably, the introduction of cγAAs as well as cβAAs and dαAAs into the new libraries resulted in the discovery of a novel binding motif.

In addition, we investigated the cellular activity of macrocyclic l-α/d-α/β/γ-hybrid peptides that showed strong in vitro inhibitory activity and prolonged serum stability. The tested peptides (IB1Orn, IB4γ^2^13γ^1^Orn, and IB5γ^2^11γ^1^) exhibited potent neutralizing activity against IFN-γ stimulation in the reporter cell line (IC_50_ = 3.5, 0.75, and 1.4 μM, respectively). This indicates that the peptides are sufficiently selective and stable to retain their activity in a cellular environment, which is more complex compared to in vitro conditions. Notably, to the best of our knowledge, this is the first demonstration that de novo macrocyclic peptides with cβAAs or cγAAs can effectively modulate the PPI at a cellular level, highlighting the potential of this class of molecules as a promising source of drug candidates.

Despite the advantages of hybrid peptides as promising drug candidates, only a few examples of bioactive α/β/γ-hybrid peptides reported to date.^58−60^ This can be attributed to the difficulties in efficiently preparing a peptide library that is applicable for high-throughput screening. Here, we successfully prepared macrocyclic l-α/d-α/β/γ-hybrid peptide libraries utilizing the devised FIT system using the combination of engineered tRNA^Pro1E2^ and EF-P. Considering that peptides containing d-α-, β-, and/or γ-amino acids exhibit distinct secondary structures compared to α-peptides, our peptide libraries combining these diverse peptides make it possible to explore chemical spaces inaccessible via conventional methods. While the integration of various nonproteinogenic amino acids enhances the diversity of the main chain structure of peptides, there is a potential concern regarding the reduction in the variety of α-amino acids, potentially leading to diminished side chain diversity. In such cases, further promising peptides could be obtained by a two-step selection process: the initial step determines the main chain through an l-α/d-α/β/γ-hybrid peptide library, followed by the subsequent selection using a library with diverse side chains featuring an increased presence of α-amino acids, while retaining the necessary nonproteinogenic amino acids. This strategy holds promise for the development of therapeutic peptides targeting a broad spectrum of proteins, including PPIs, offering novel avenues for drug discovery.

## Supporting Information Available

Additional experimental results and methods (PDF)
Sequences of peptides obtained by in vitro selection, list of RNAs, and the corresponding primers (XLSX)
