# Supplementary material for: In Vitro Selection of Macrocyclic l-α/d-α/β/γ-Hybrid Peptides Targeting IFN-γ/IFNGR1 Protein–Protein Interaction
Source: J Am Chem Soc. 2024 Jun 18;146(26):17691–9. doi: 10.1021/jacs.4c01979 (PMC11229689; doi:10.1021/jacs.4c01979)
Supplement: Supplementary file 1 — ja4c01979_si_001.pdf [file ja4c01979_si_001.pdf]

Supporting information and experimental section for:

In vitro selection of macrocyclic L-α/D-α/β/γ-hybrid peptides targeting IFN-γ/IFNGR1 protein-protein interaction

Takashi Miura, Kang Ju Lee, Takayuki Katoh, and Hiroaki Suga*

Department of Chemistry, Graduate School of Science, The University of Tokyo

Table of Contents

**Title**	**Page**
Figure S1. Secondary structures of tRNAs used for incorporation of nonproteinogenic amino acids.	S3
Figure S2. Design of previously reported library (library P).	S4
Figure S3. RaPID selection against IFNGR1 using ribosomally synthesized macrocyclic peptide libraries.	S5
Figure S4. Structures of selected macrocyclic L-α/D-α/β/γ-hybrid peptides.	S6
Figure S5. Identities of peptides synthesized by SPPS.	S8
Figure S6. Binding kinetics of macrocyclic peptides against IFNGR1.	S10
Figure S7. Inhibitory activity of macrocyclic peptides against IFN-γ/IFNGR1 PPI determined by AlphaLISA.	S13
Figure S8. Serum stability assay of macrocyclic peptides.	S14
Figure S9. Cell viability assay of IB1Orn, IB4$\gamma^2$13γ^1Orn, and IB5$\gamma^2$11γ^1 on HEK-Dual^{TM} cells.	S36
Materials and methods	S37
References for Supporting Information	S41

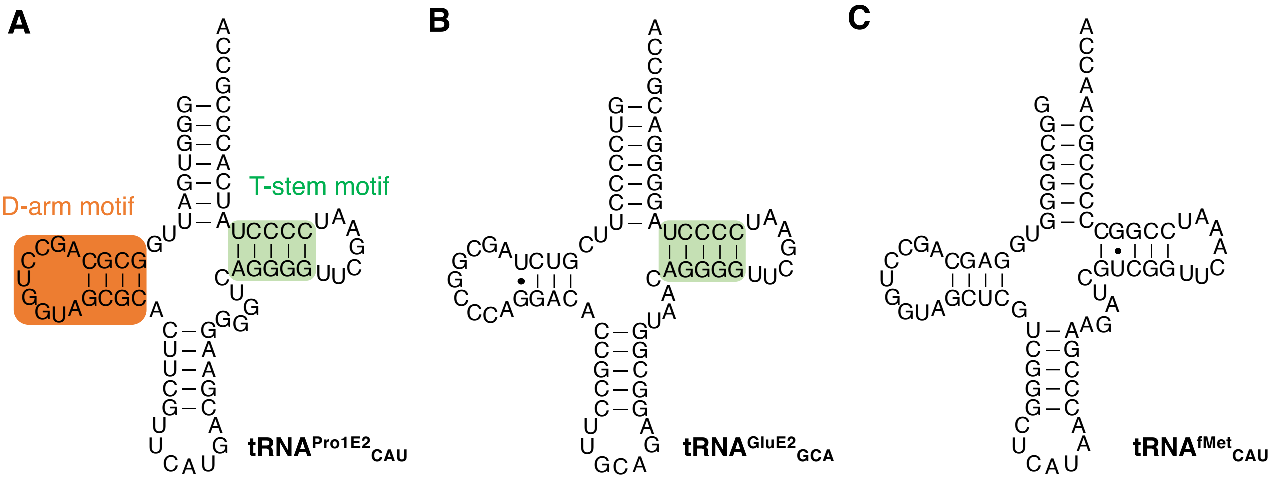

Figure S1. Secondary structures of tRNAs used for incorporation of nonproteinogenic amino acids. (A) tRNA$^{Pro1E2}{}_{CAU}$ for suppression of AUG codons. For decoding other codons, the sequence of anticodon loop was changed accordingly (see SI Table S2 for the sequences). The D-arm motif for EF-P binding consists of a 9-nt D-loop closed by a stable 4-bp stem (highlighted in orange). The T-stem motif for EF-Tu binding is highlighted in green. (B) tRNA$^{GluE2}{}_{GCA}$ used for suppression of UGC codons. The T-stem motif for EF-Tu binding highlighted in green is identical to that of tRNA^{Pro1E2}. (C) tRNA$^{fMet}{}_{CAU}$ used for incorporation of ^{ClAc}y at the initiator AUG codon.

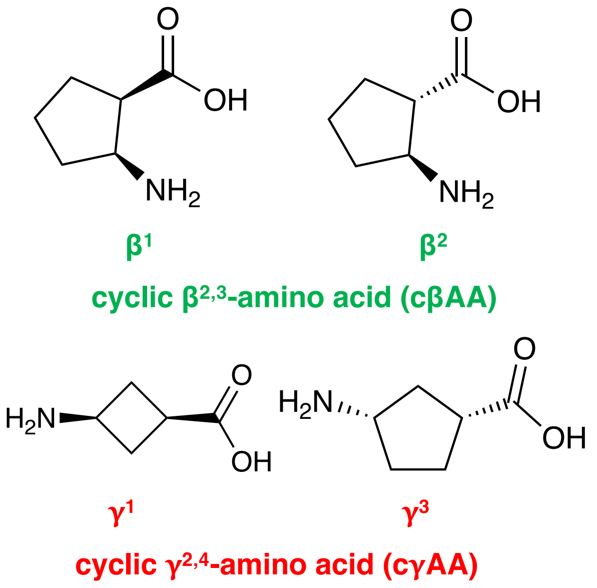

1st	2nd				3rd
	U	C	A	G	
U	F	S	Y	β^2	U C
	L	S	stop		A G
C	L	P	H	R	U C
	L	P		R	A G
A	I	T	N	S	U C
	I	T	K	R	A
	^{ClAc}y / c				G
G	β^1	A	D	G	U C
		A		G	A
	γ^3		γ^1		G

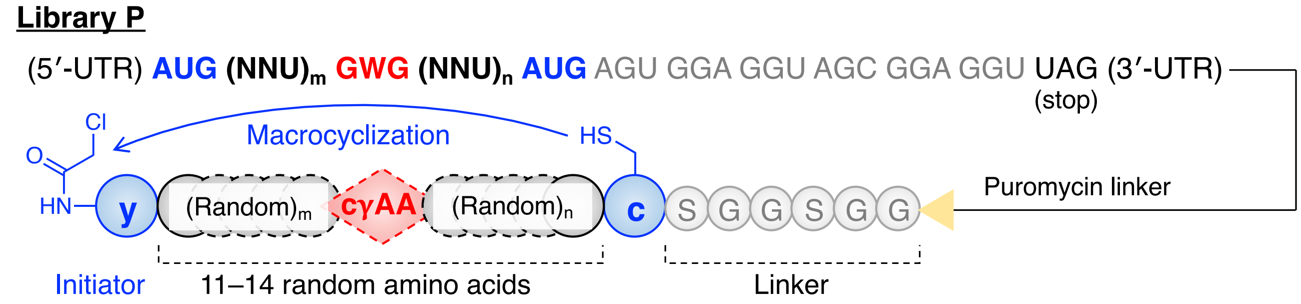

Figure S2. Design of previously reported library (library P)[1]**.** Two cβAAs, (1*R*,2*S*)-2-aminocyclopentane carboxylic acid (β^1) and (1*S*,2*S*)-2-aminocyclopentane carboxylic acid (β^2), and two cγAAs, *cis*-3-aminocyclobutane carboxylic acid (γ^1) and (1*R*,3*S*)-3-aminocyclopentane carboxylic acid (γ^3) were assigned at GUU, UGU, GAG, and GUG codons. For macrocyclization of peptide, *N*-chloroacetyl-D-tyrosine (^{ClAc}y) and D-cysteine (c) were introduced at the initiator and elongator AUG codons. The peptide library comprised a repeat of 11 – 14 random residues encoded by GWG and NNU codons (W = A or U; N = A, U, G or C) flanked by the cyclizing ^{ClAc}y and c residues.

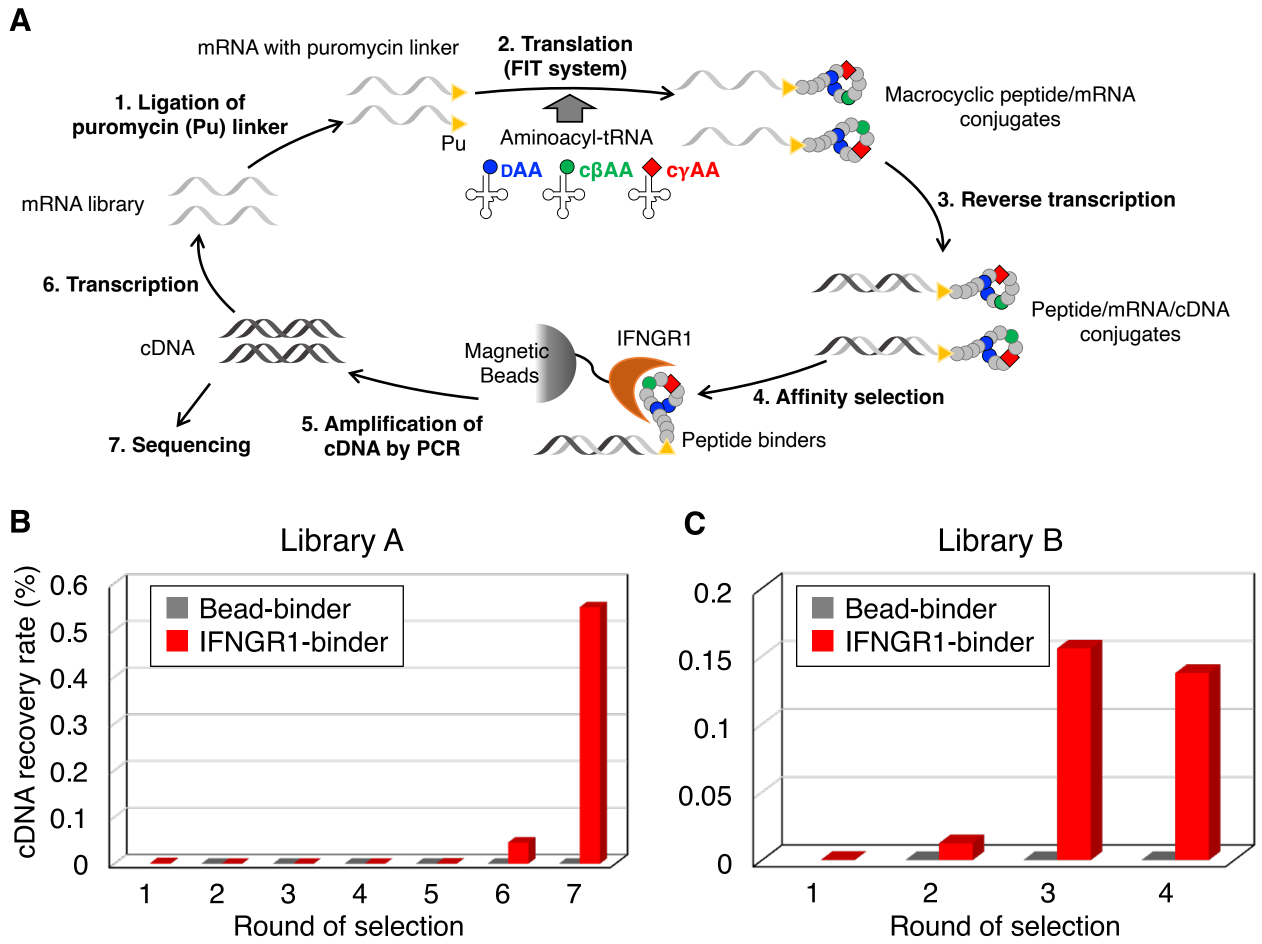

Figure S3. RaPID selection against IFNGR1 using ribosomally synthesized macrocyclic peptide libraries. (A) Schematic depiction of RaPID selection. 1) Ligation of puromycin linker to the 3′-end of the mRNA library. 2) Translation of peptides using the reprogrammed genetic code with nonproteinogenic aminoacyl-tRNAs, followed by macrocyclization of peptide between ^{ClAc}y and c via a thioether bond. 3) Reverse transcription of mRNA into cDNA. 4) Affinity selection of peptides against naked magnetic beads, followed by selection against IFNGR1 immobilized beads. 5) Recovery of the bound fraction and amplification of cDNA of binders by PCR. 6) Transcription of cDNA into mRNA. 7) Next generation sequencing analysis of cDNA. (B,C) Recovery rate of cDNA after the affinity selection at each round using libraries A and B, respectively. Red and gray bars indicate the recovery rate of IFNGR1 binders and beads binders, respectively. Bead-binder selection was not performed in the first round.

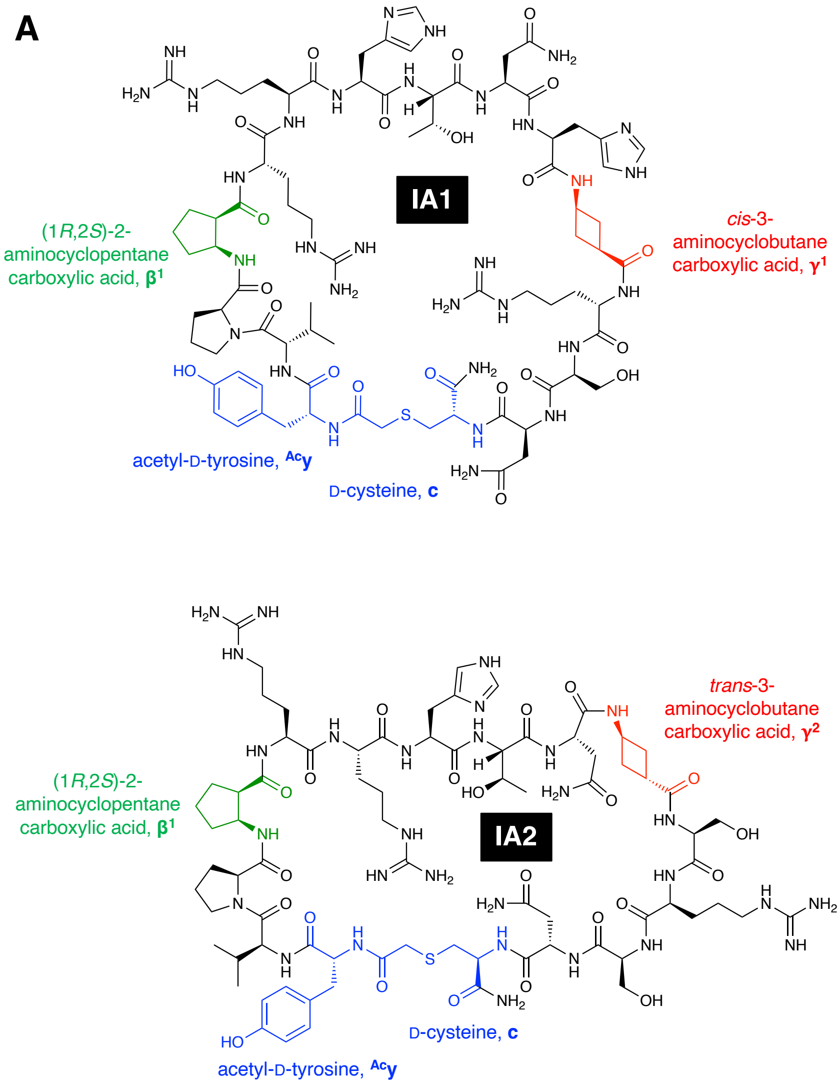

Figure S4. Chemical structures of selected macrocyclic L-α/D-α/β/γ-hybrid peptides. (A) Structures of IA1 and IA2. (B) Structures of IB1−5. See Figure 4 for ornithine variants, IB1Orn and IB4$\gamma^2$13γ^1Orn.

B

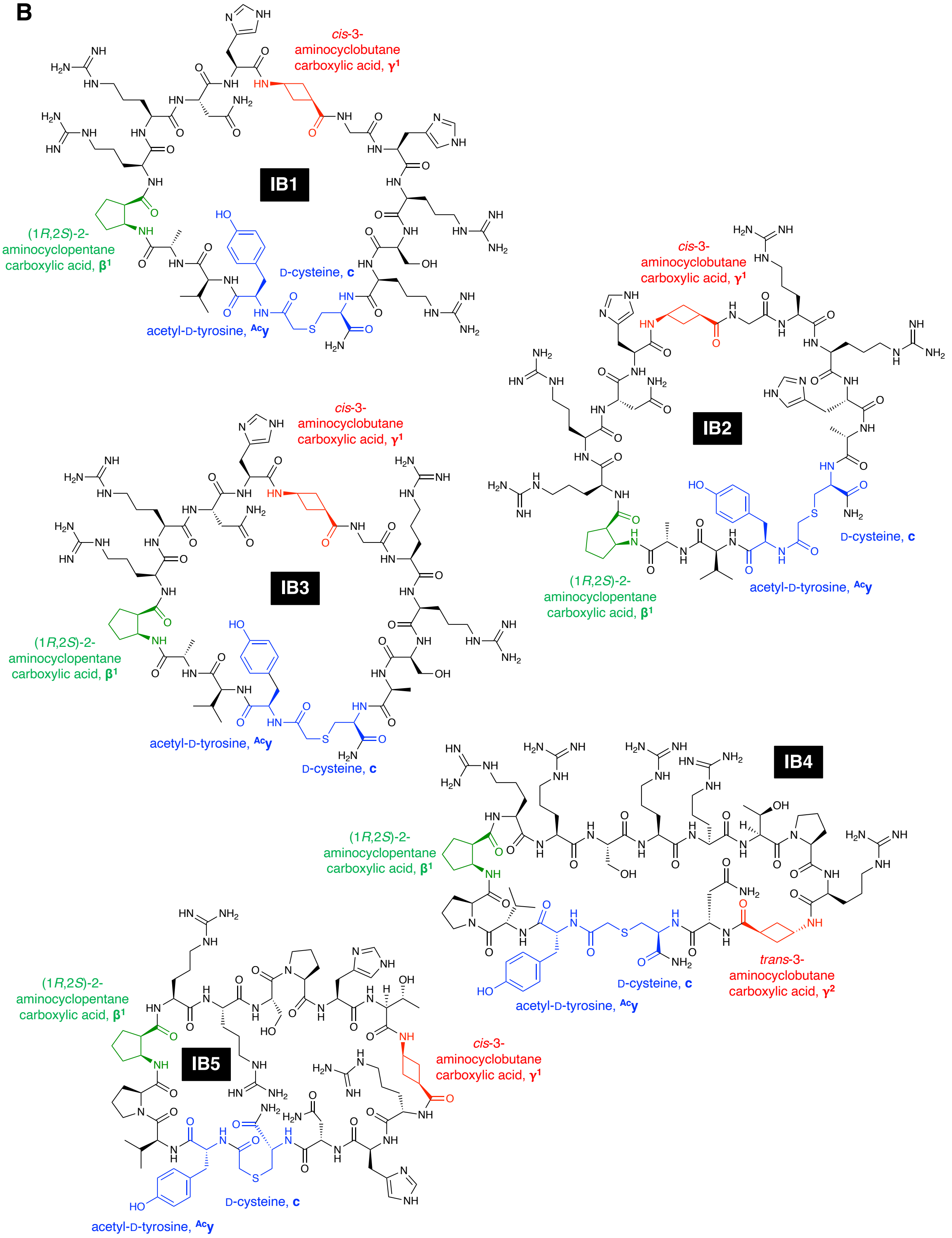

Figure S4, continued.

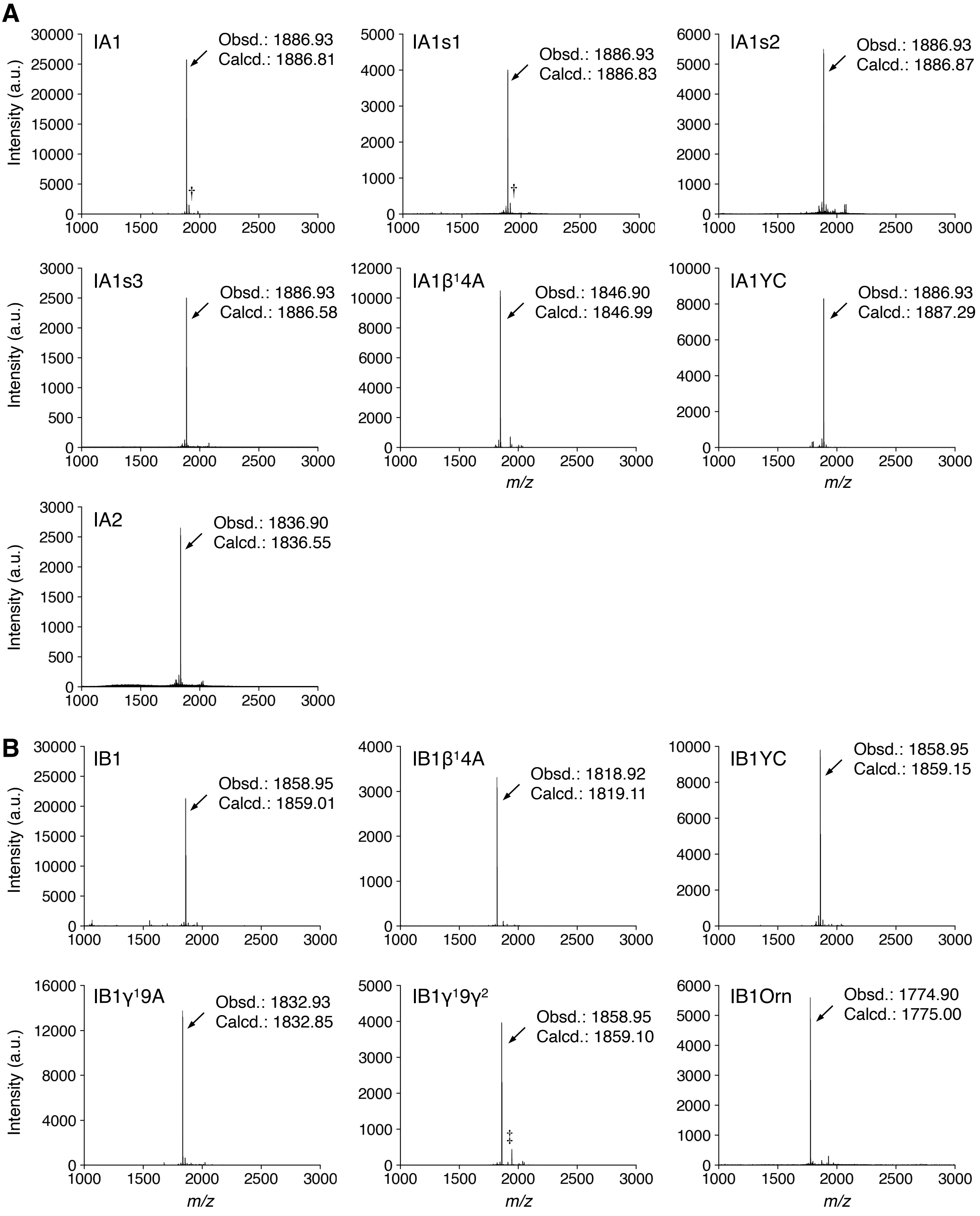

Figure S5. Identities of peptides synthesized by SPPS. (A) MALDI-TOF mass spectra of IA1, IA2 and their variants. (B,C) IB1−5 and their variants. Calcd. and Obsd. indicate calculated and observed $[M+H]^+$ values, respectively. † and ‡ indicate sodium and potassium ion adducts, respectively.

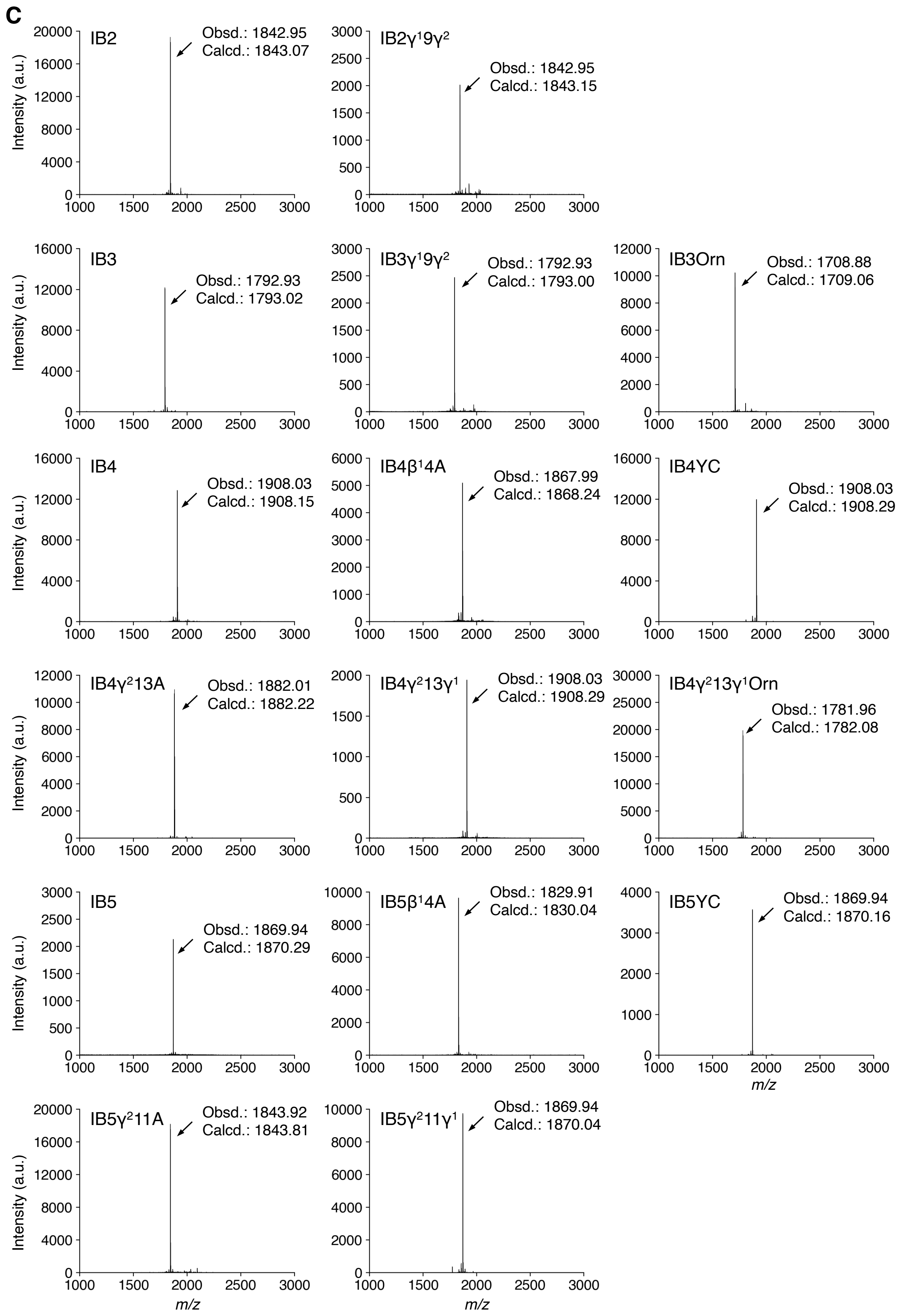

Figure S5, continued.

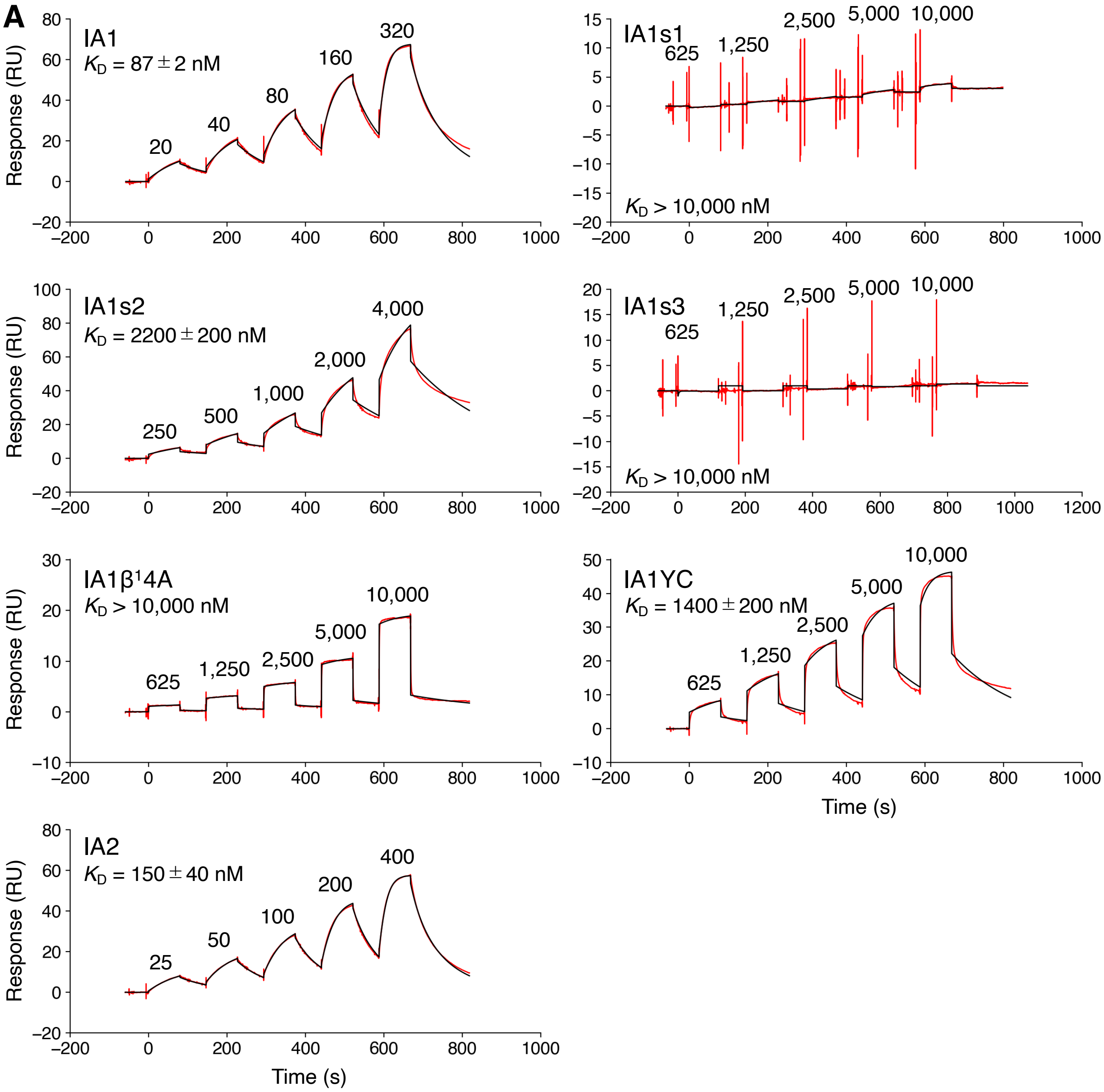

Figure S6. Binding kinetics of macrocyclic peptides against IFNGR1. SPR sensorgrams of selected peptides and variants. (A) IA1, IA2, and their variants. (B,C) IB1−5 and their variants. The sequences and kinetic values are shown in Table 1. Five different concentrations (nM), indicated by the numbers above the sensorgrams, of each peptide were injected for measuring kinetic constants. Binding sensorgrams were fitted using the standard 1:1 binding model. Red and black lines indicate raw sensorgrams and fitted curves, respectively.

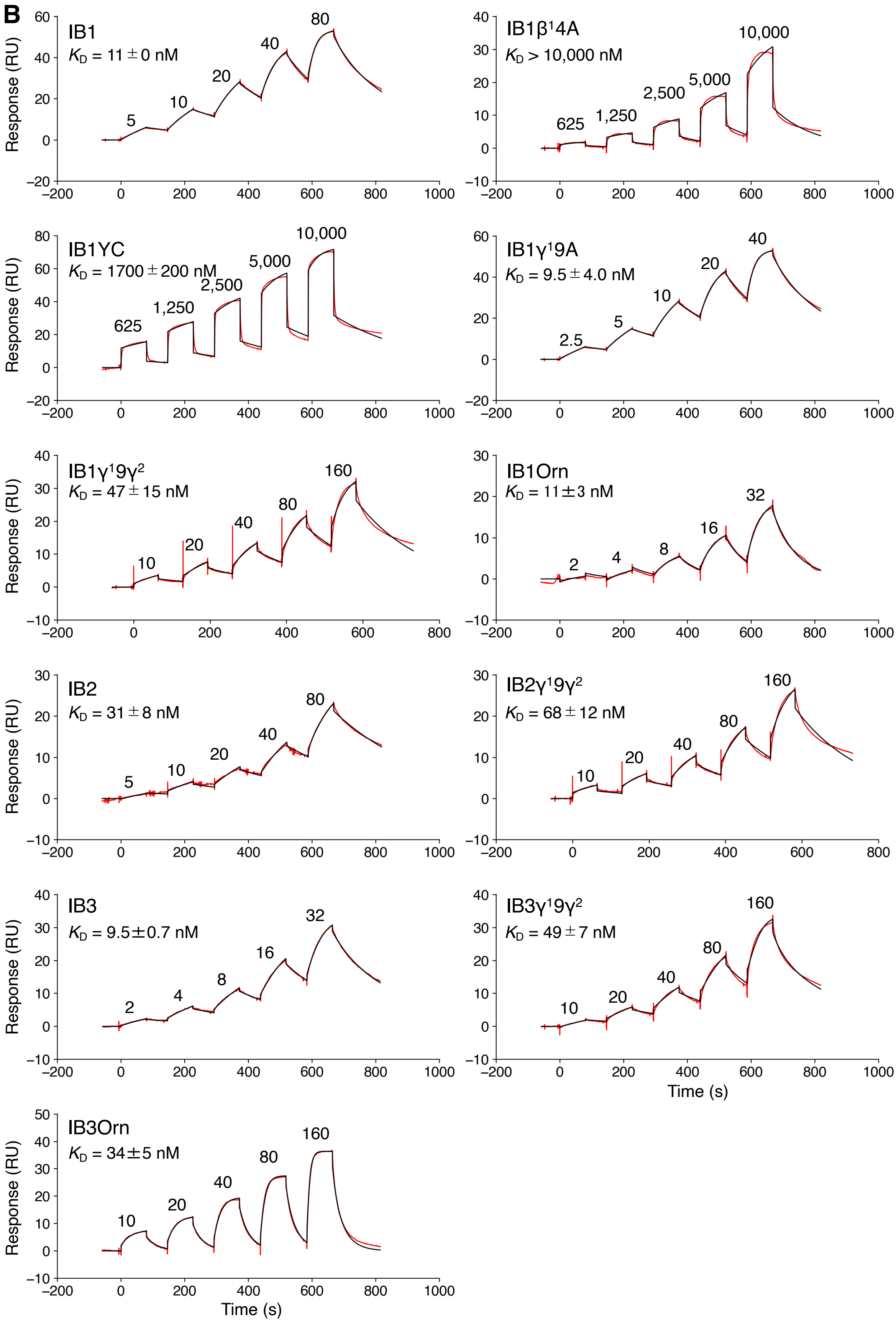

Figure S6, continued.

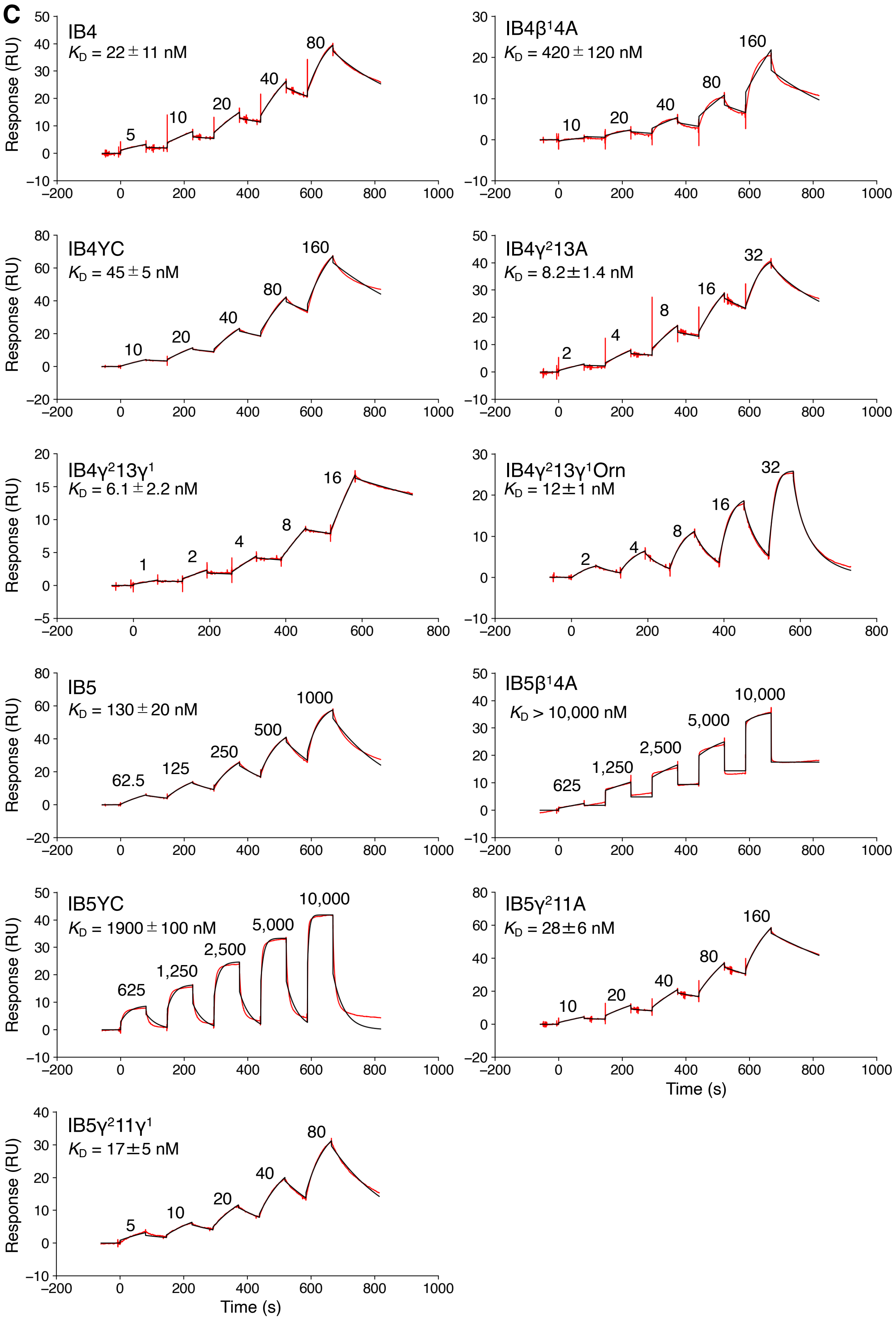

Figure S6, continued.

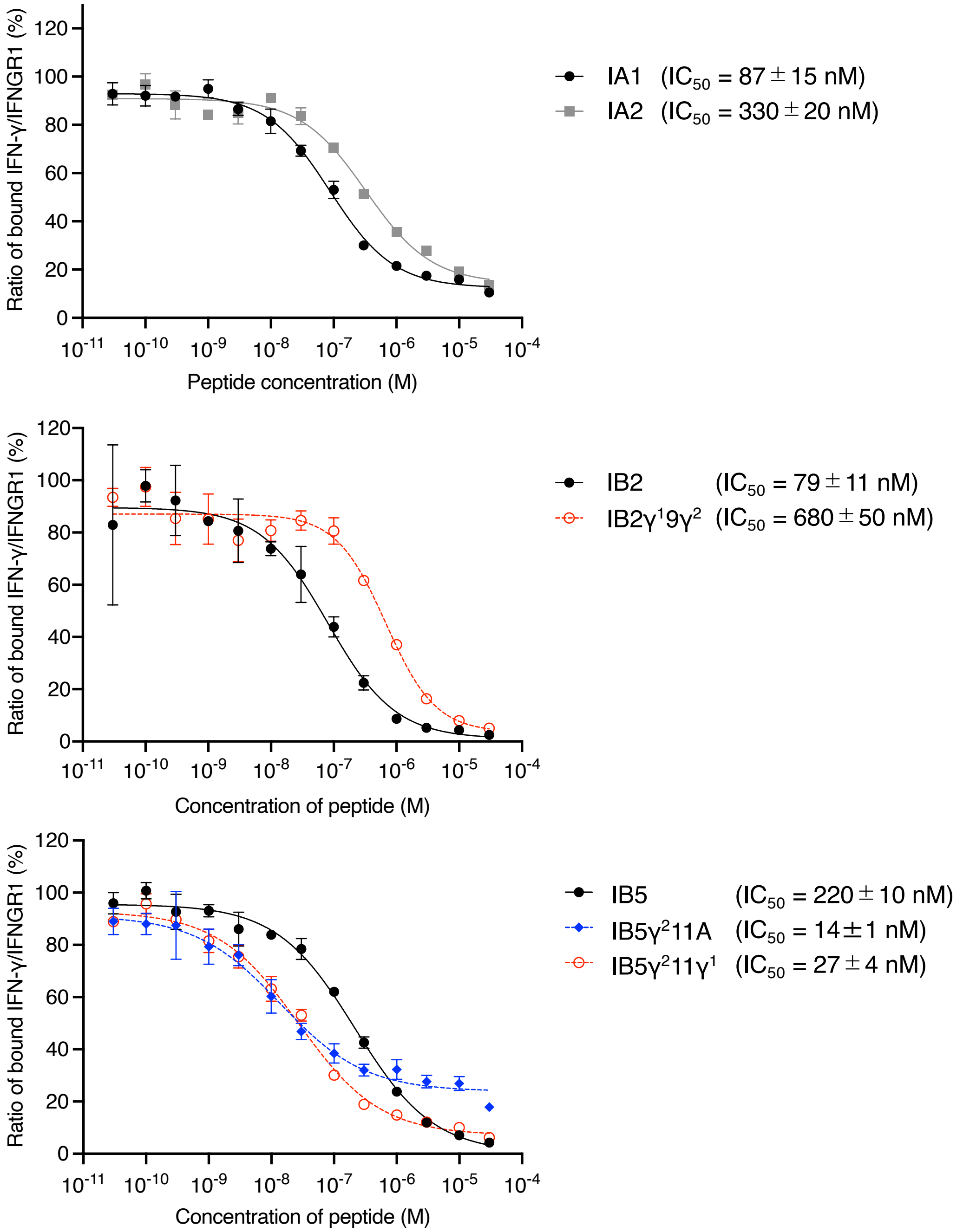

Figure S7. Inhibitory activity of IA1, IA2, IB2, IB5, and their variants against IFN-γ/IFNGR1 PPI determined by AlphaLISA. Data are presented as mean values ± standard deviation, SD (n = 3). IC_{50} value was estimated by a four-parameter dose-response curve fitting using GraphPad Prism 9. See Figure 2 for IB1, IB3, IB4, and their variants.

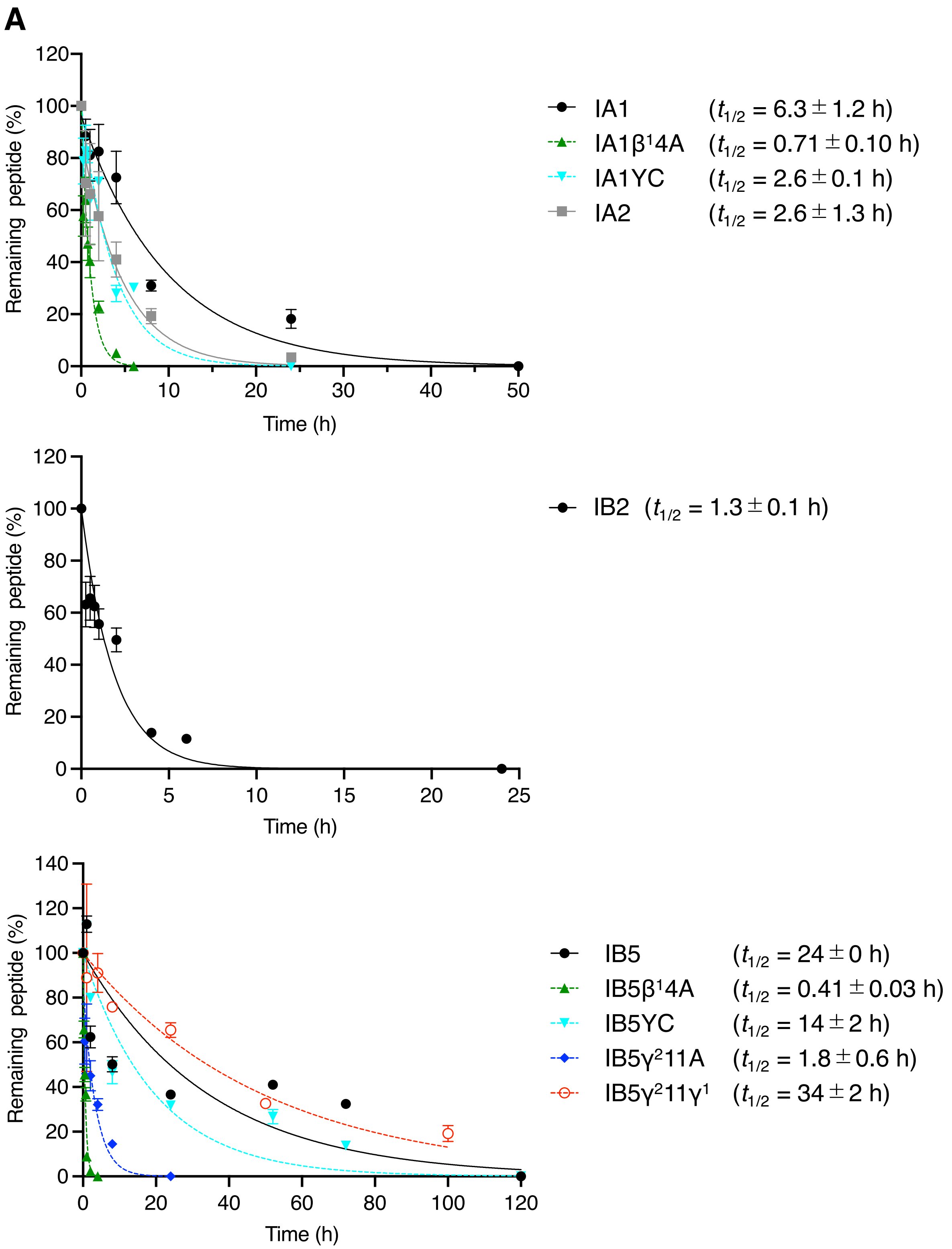

Figure S8. Serum stability assay of macrocyclic peptides. (A) Degradation of peptides in human serum. Results of IA1, IA2, IB2, IB5, and their variants are shown. See Figure 3 for IB1, IB3, IB4, and their variants. Each peptide was co-incubated with an internal standard peptide in human serum at 37 °C. At each time point, the relative intensity of each peptide to the standard peptide were estimated by LC/MS. Half-life of peptide ($t_{1/2}$) was analyzed by non-linear regression curve fitting using

GraphPad Prism 9. Data are presented as mean ± SD (n = 3). (B−D) Fragments of IB1 (IB1-f1−5), IB1Orn (IB1Orn-f1−11), IB3 (IB3-f1−6), IB3Orn (IB3Orn-f1−12), IB4 (IB4-f1−8), IB4$\gamma^2$13γ^1 (IB4$\gamma^2$13γ^1f1−f4), IB4$\gamma^2$13γ^1Orn (IB4$\gamma^2$13γ^1Orn-f1−f10) IB5 (IB5-f1−4), and IB5$\gamma^2$11γ^1 (IB5$\gamma^2$11γ^1-f1−4) at timepoints near their half-lives were analyzed by LC/MS. (E−H) Chromatogram and mass spectra of reaction mixture of serum stability assay. Blue, red, and black arrows indicate $[M+3H]^{3+}$, $[M+4H]^{4+}$, and $[M+5H]^{5+}$ ions, respectively. Calcd. and Obsd. indicate calculated and observed *m/z* values. (I−T) Chromatogram and mass spectra of fragments. Black, orange, blue, red, black arrows indicate $[M+H]^{+}$, $[M+2H]^{2+}$, $[M+3H]^{3+}$, $[M+4H]^{4+}$, and $[M+5H]^{5+}$ ions, respectively. Calcd. and Obsd. indicate calculated and observed *m/z* values. (U) Sequencing of fragment peptides by LC/MS/MS. MS/MS spectra of fragments IB1-f1, IB3-f1, and IB4-f1 are shown. Observed b and y ions are indicated in blue and orange, respectively.

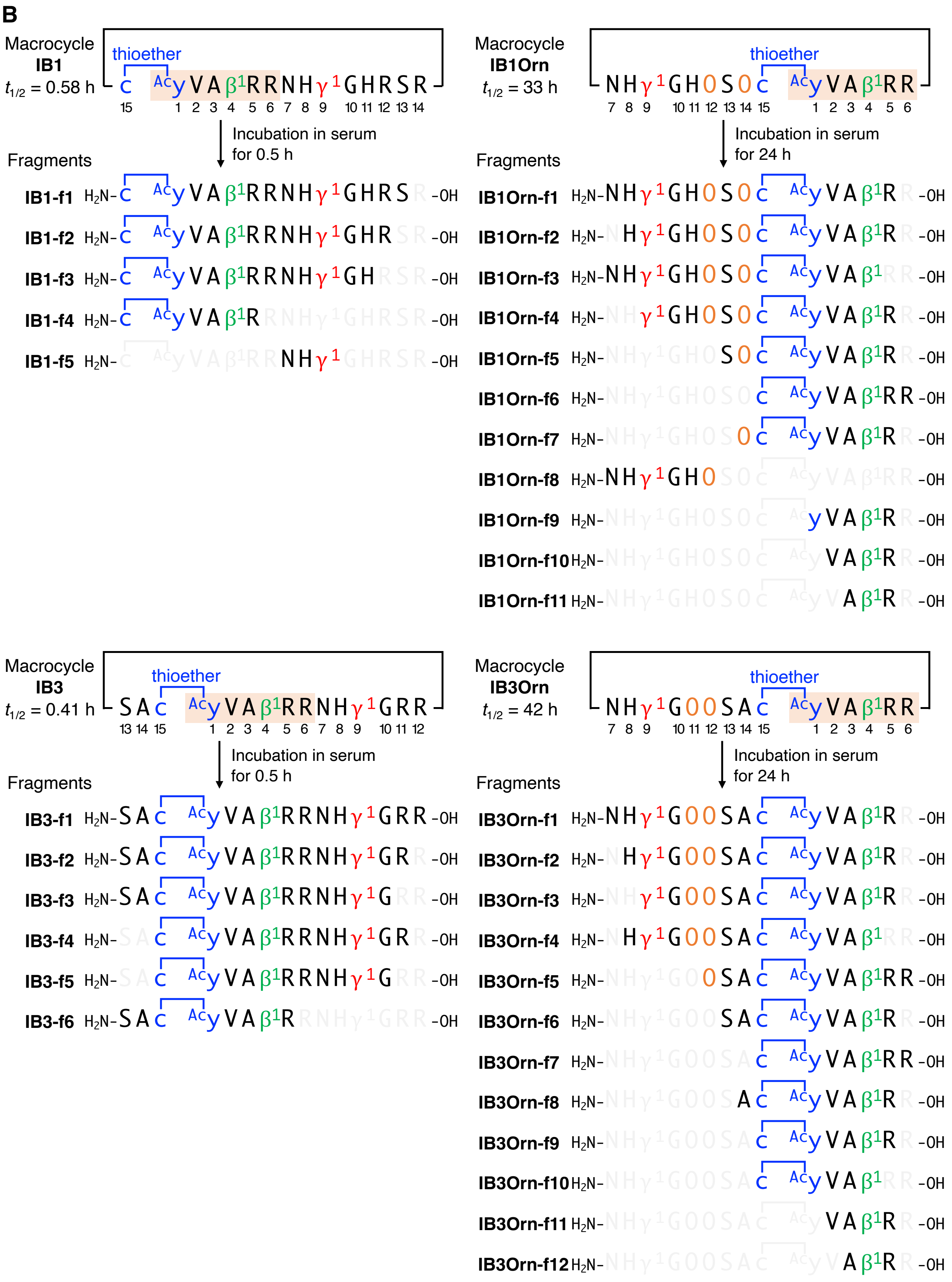

Figure S8, continued.

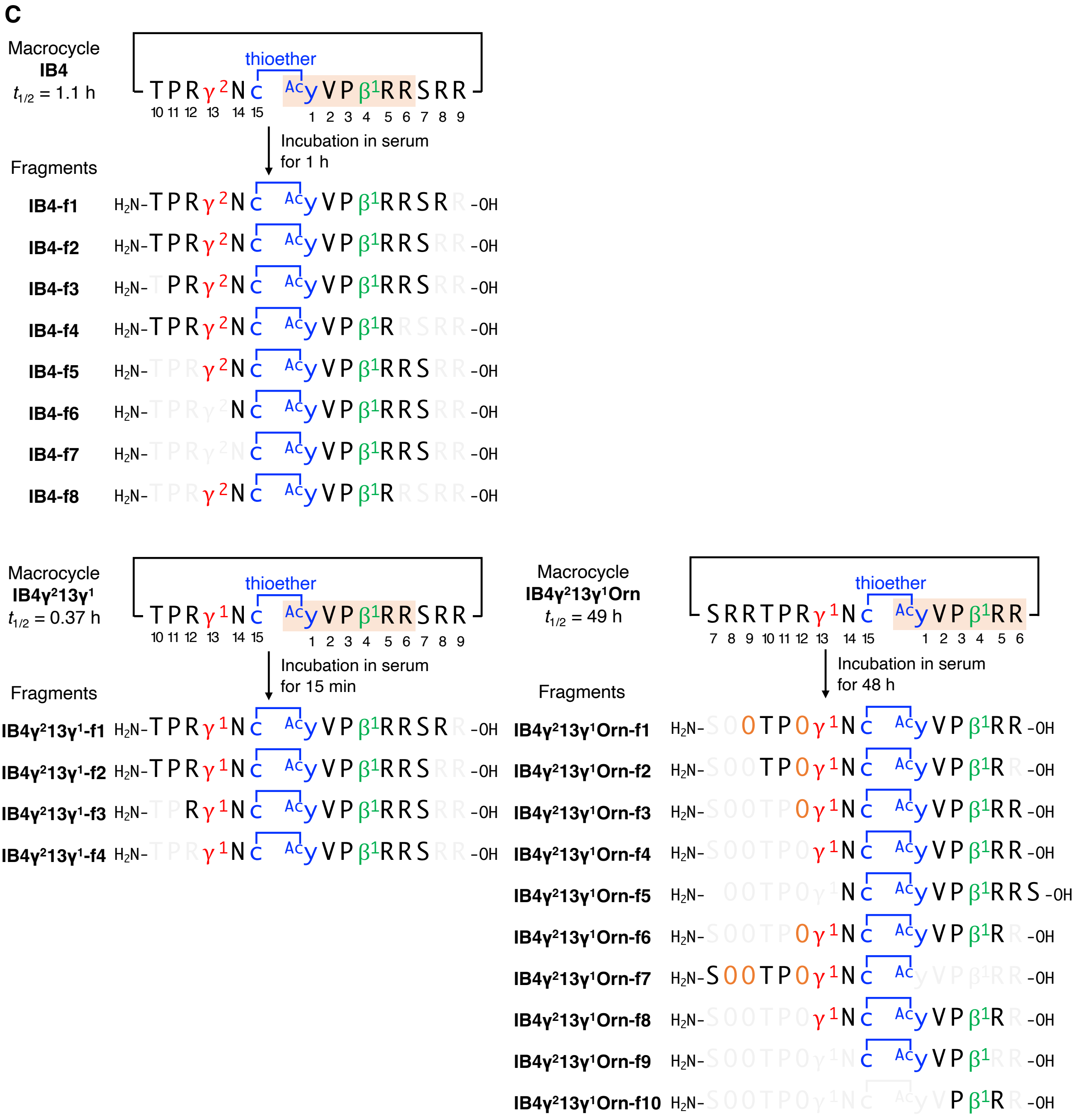

Figure S8, continued.

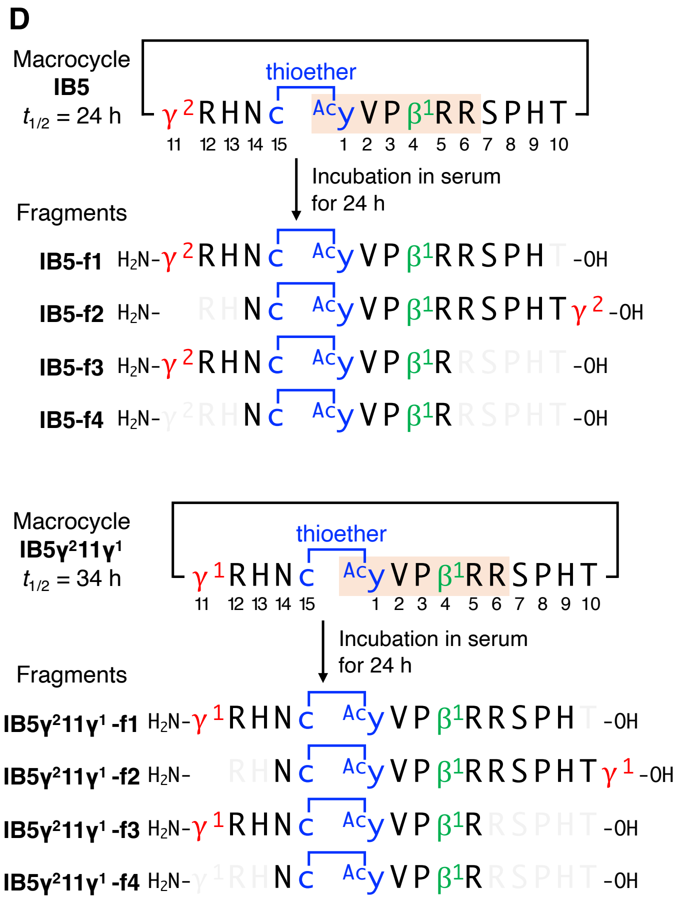

Figure S8, continued.

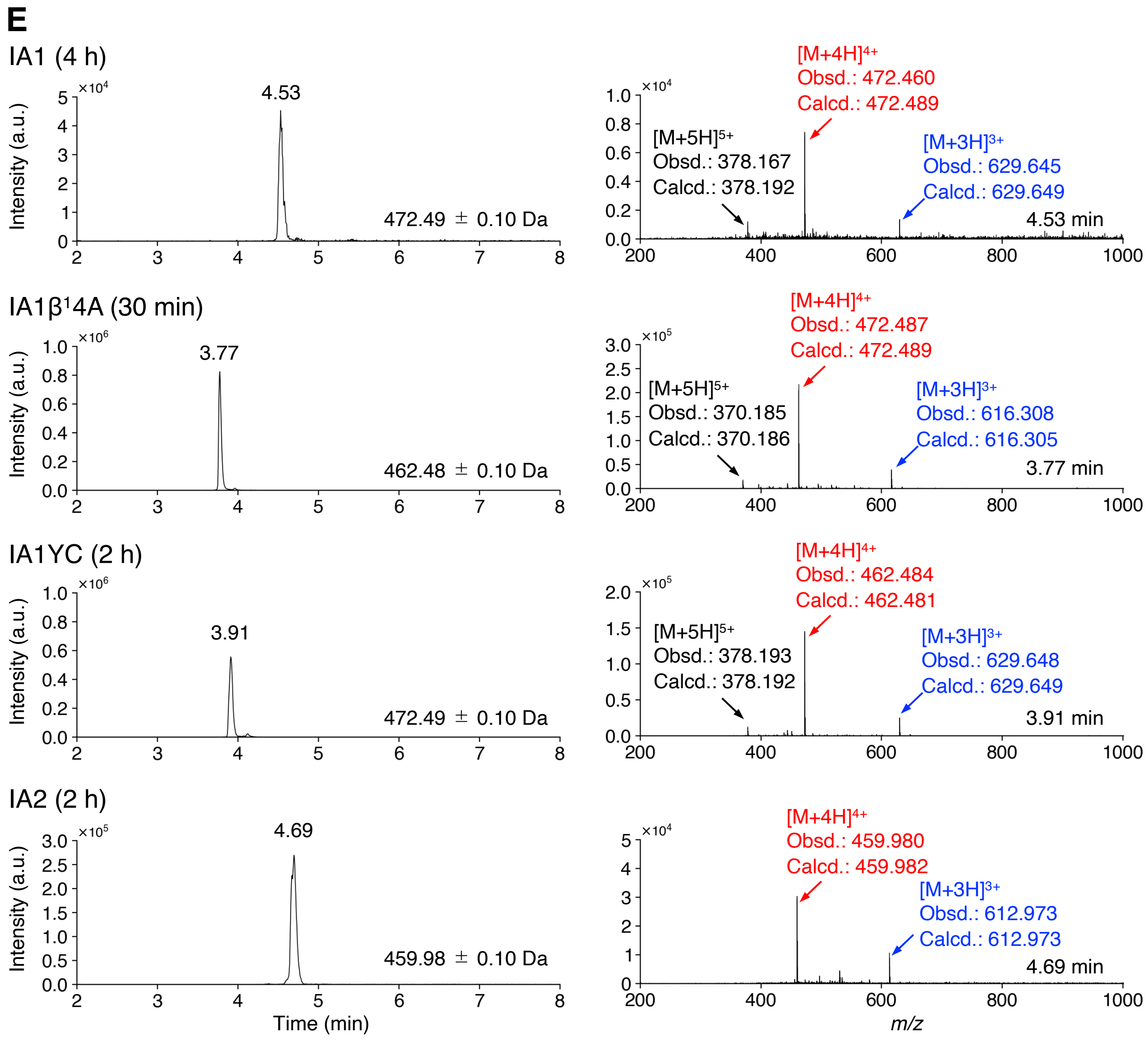

Figure S8, continued.

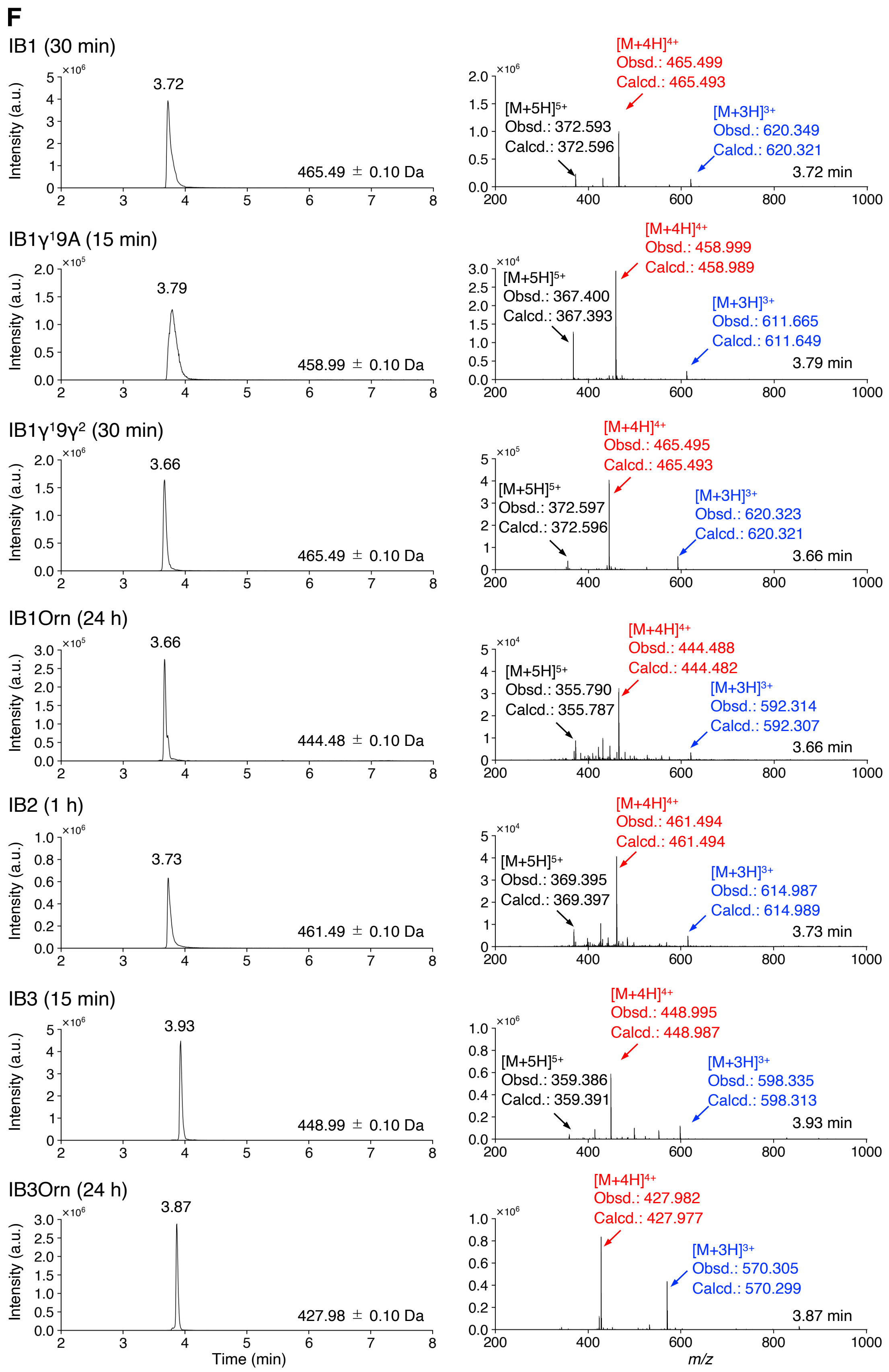

Figure S8, continued.

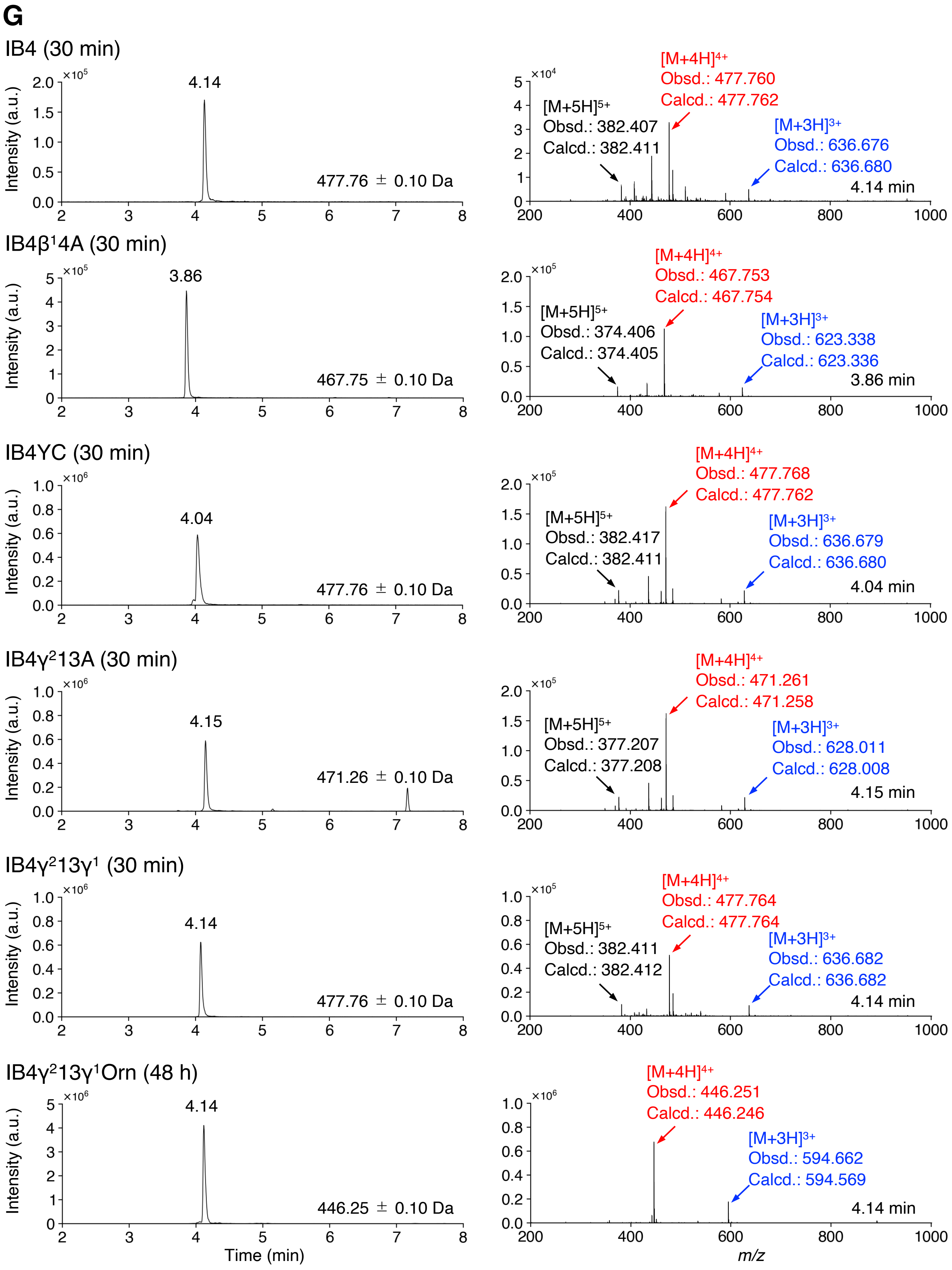

Figure S8, continued.

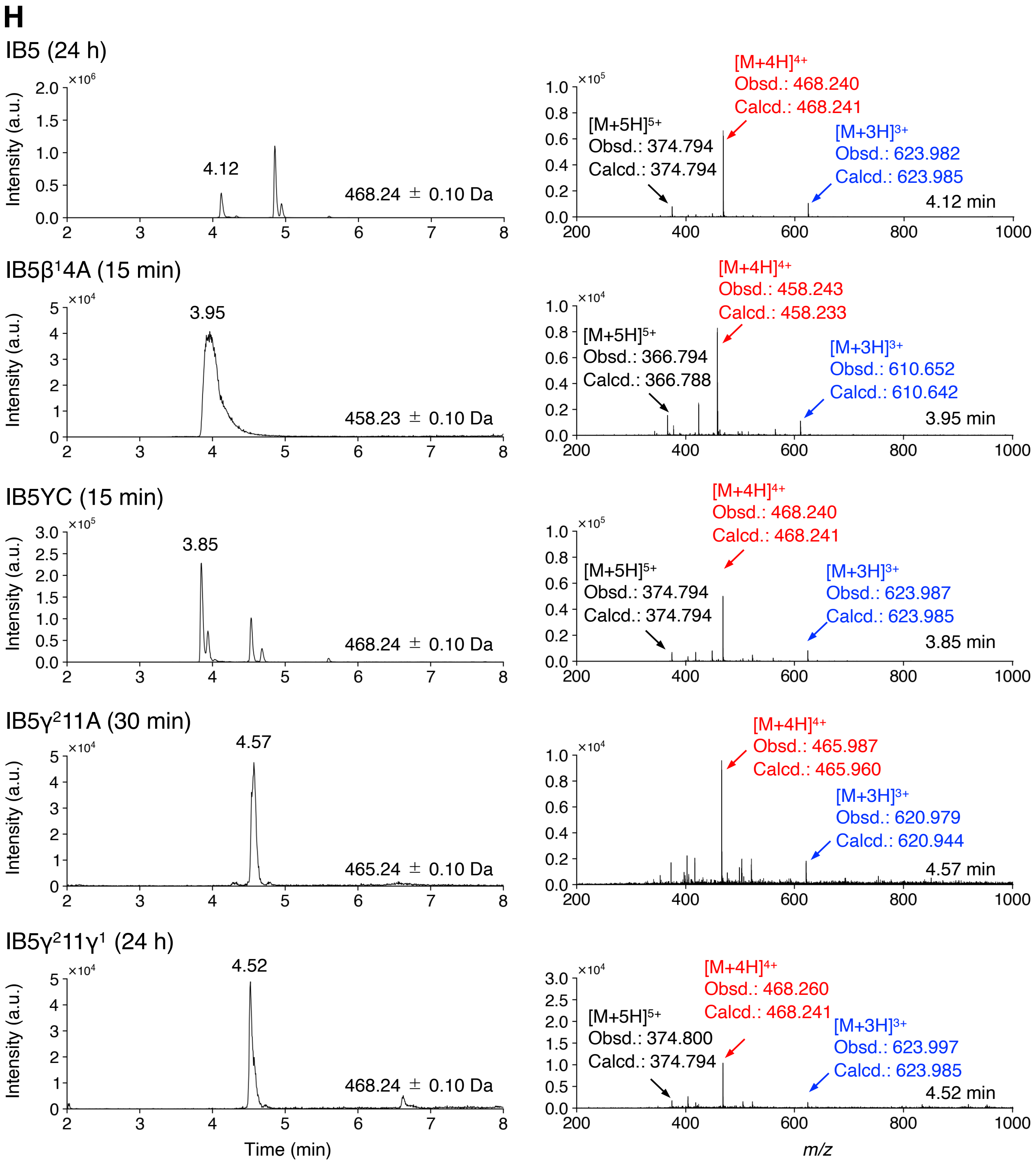

Figure S8, continued.

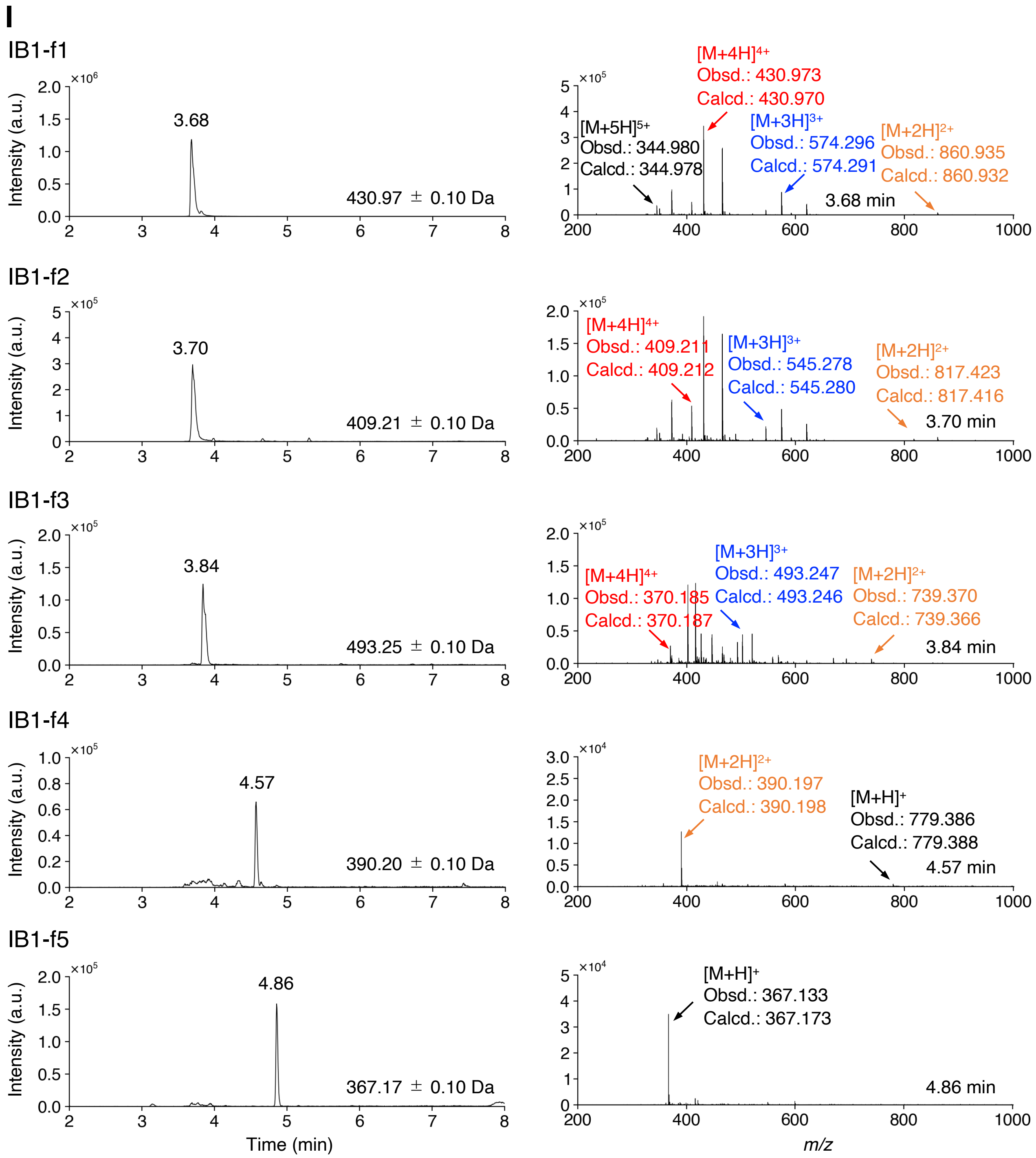

Figure S8, continued.

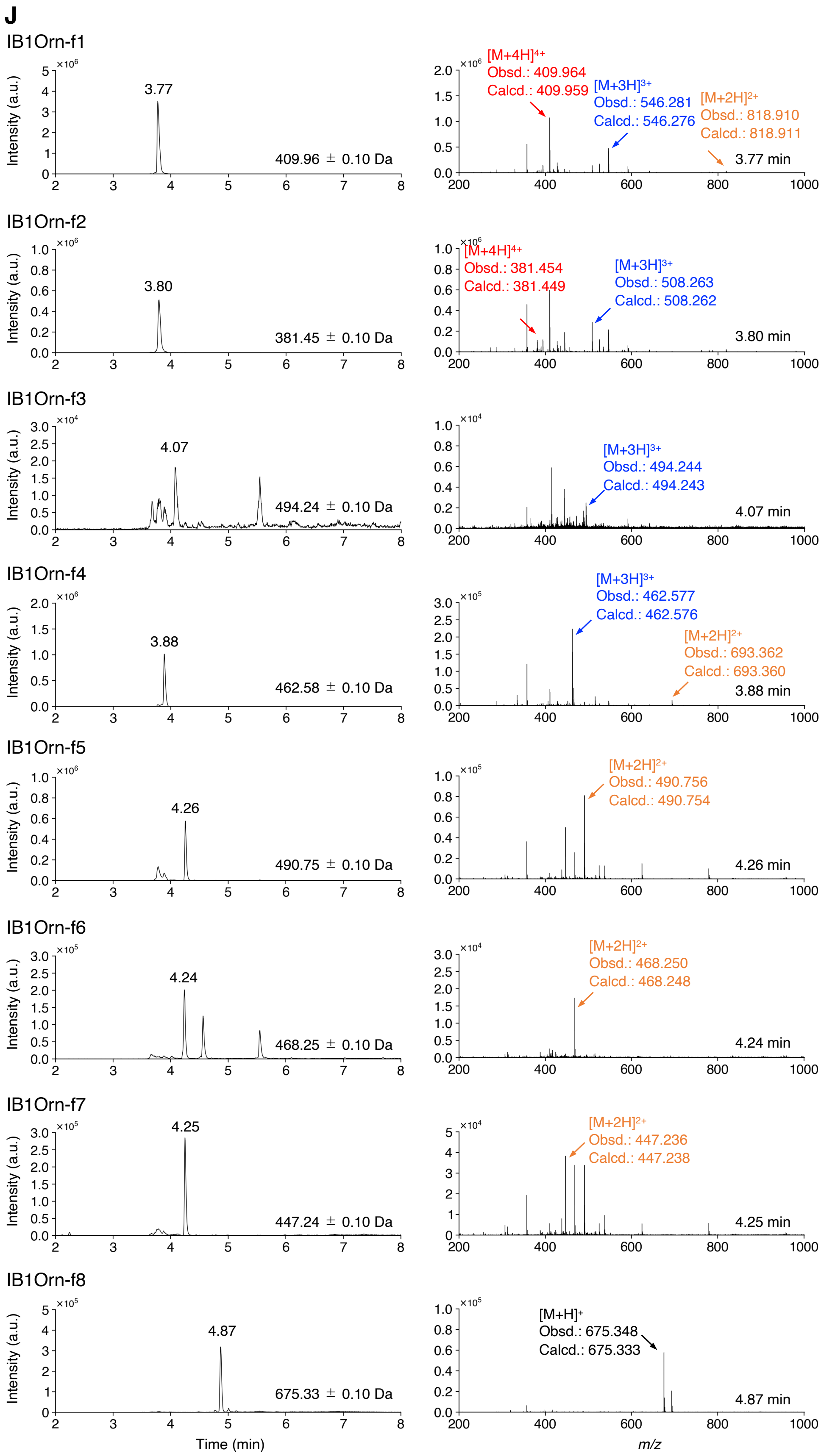

Figure S8, continued.

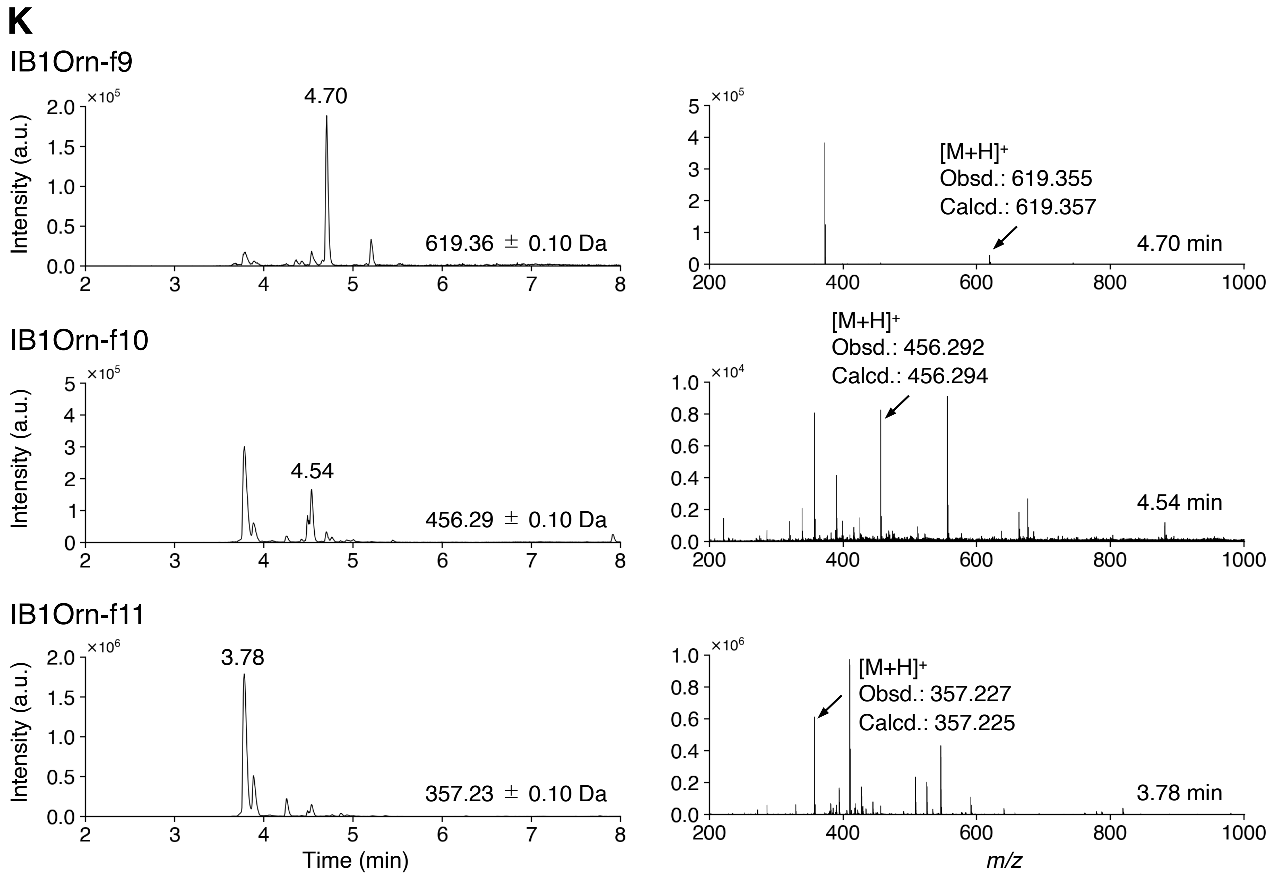

Figure S8, continued.

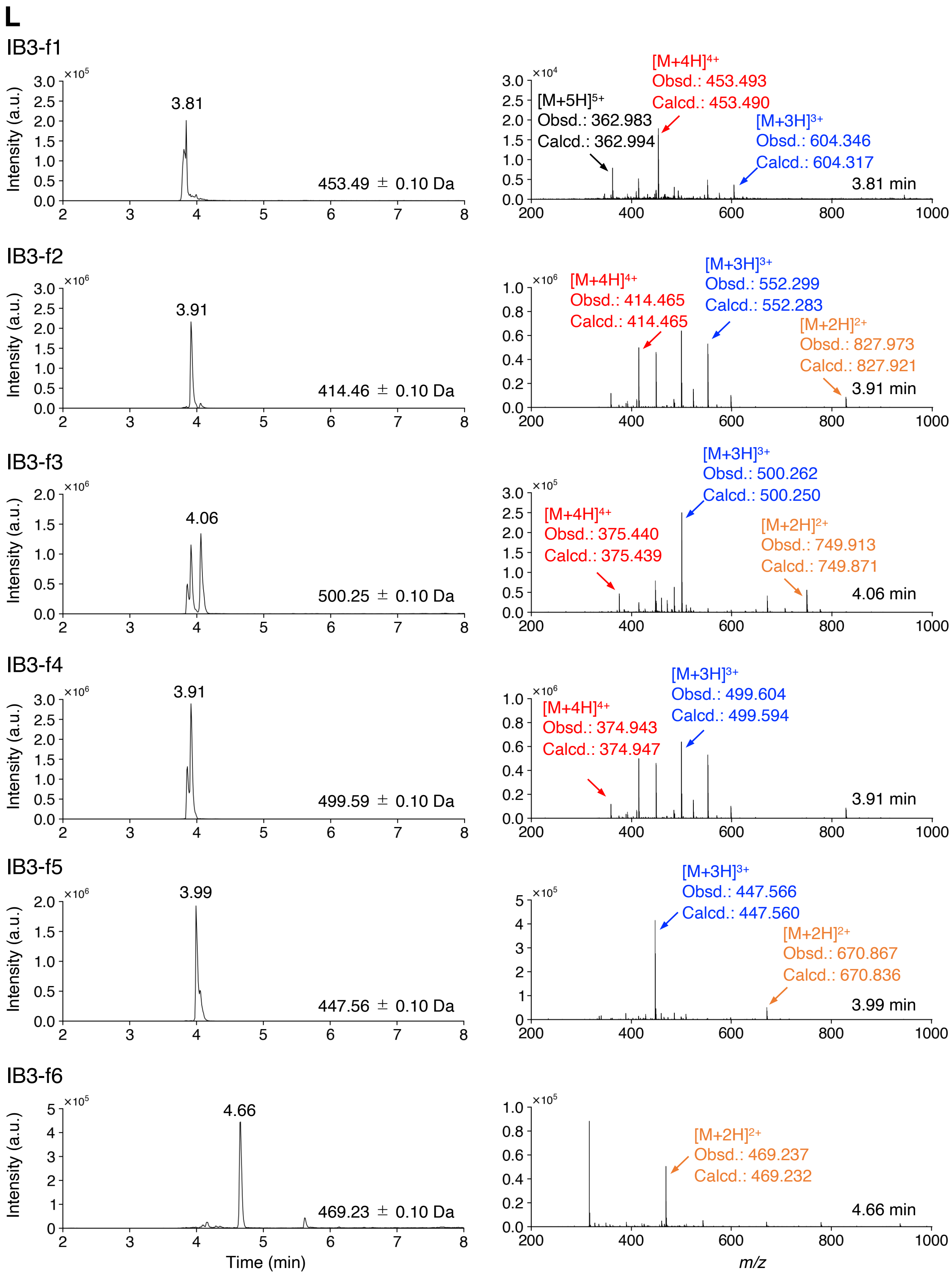

Figure S8, continued.

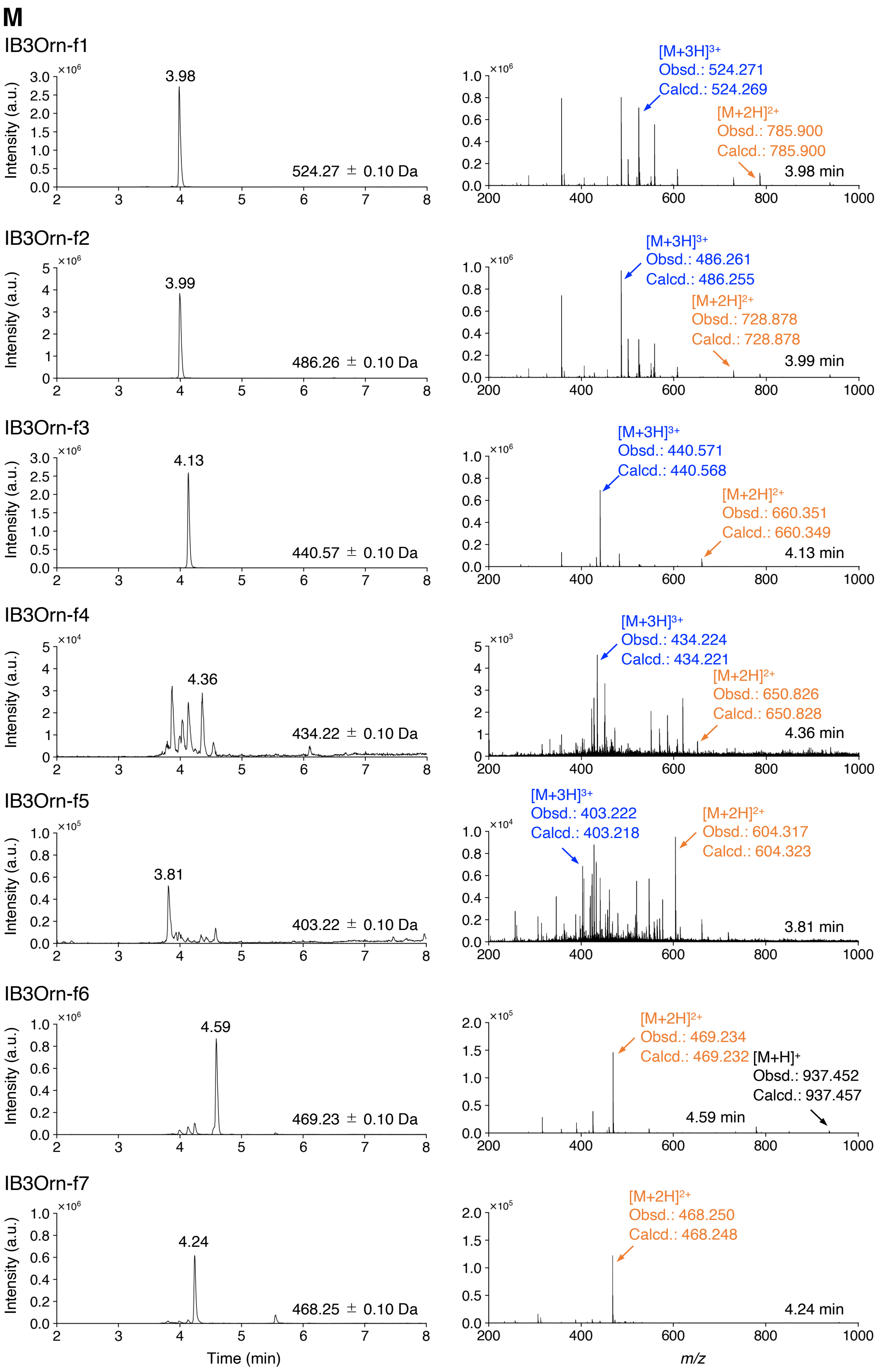

Figure S8, continued.

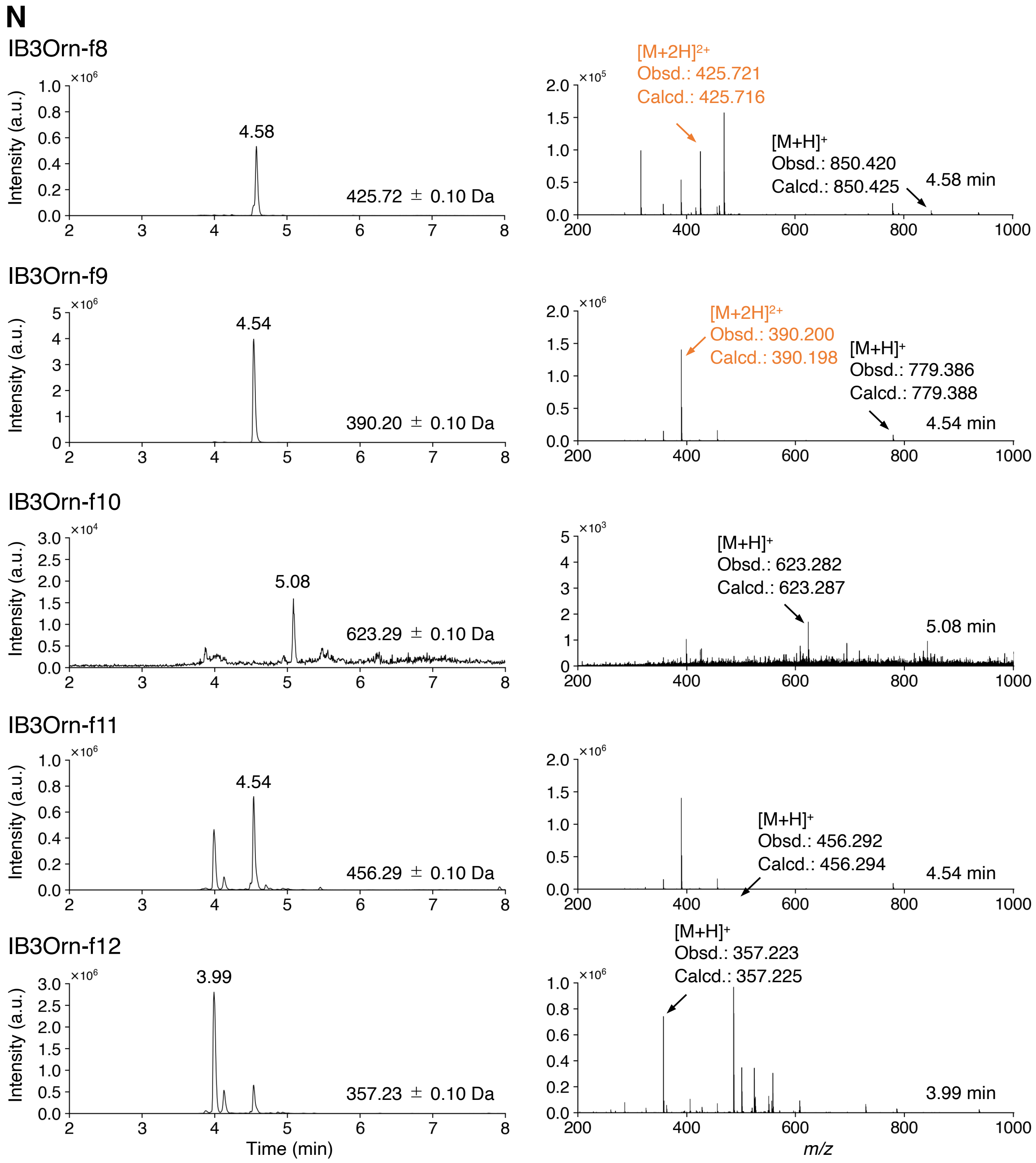

Figure S8, continued.

O

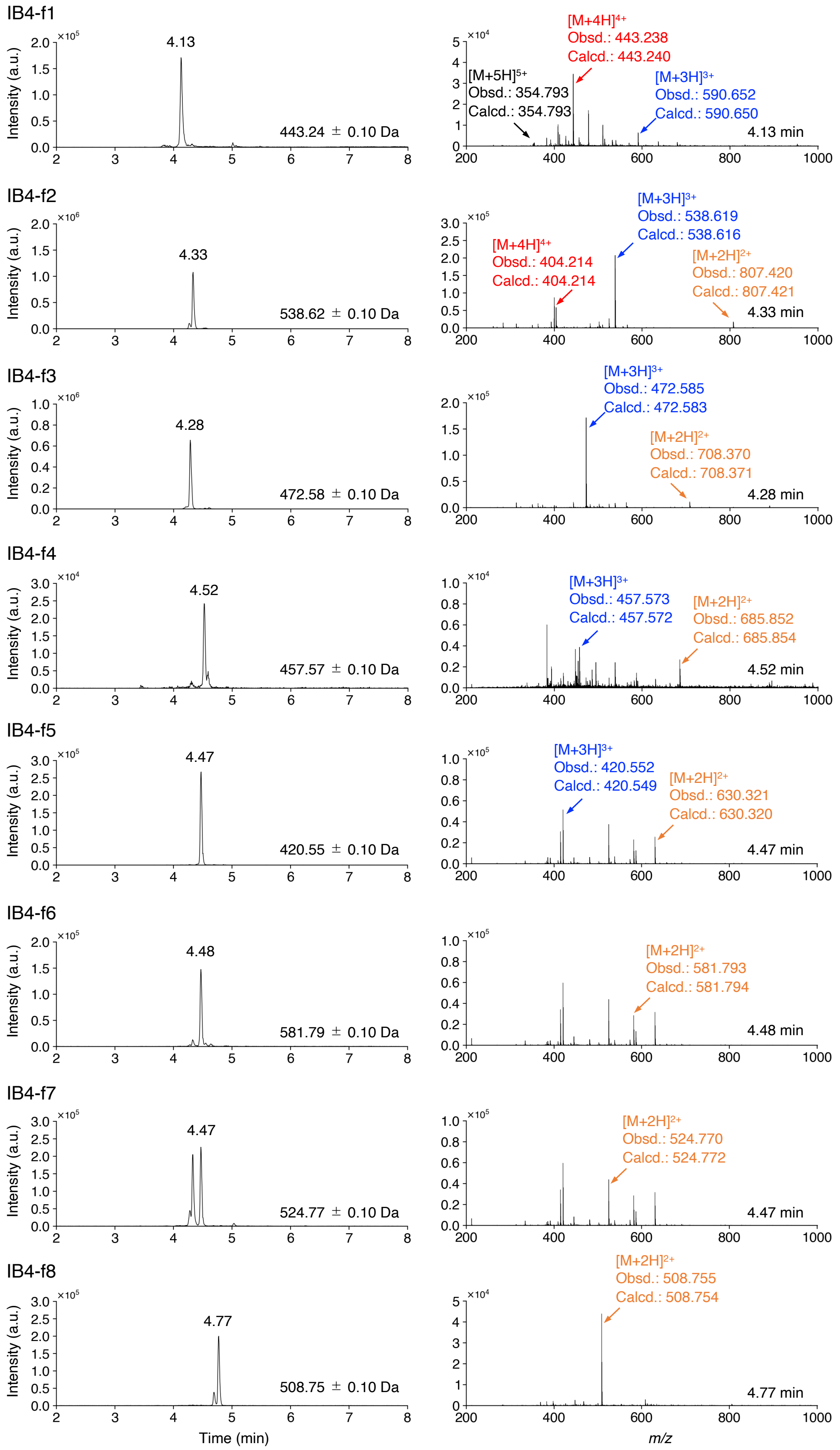

Figure S8, continued.

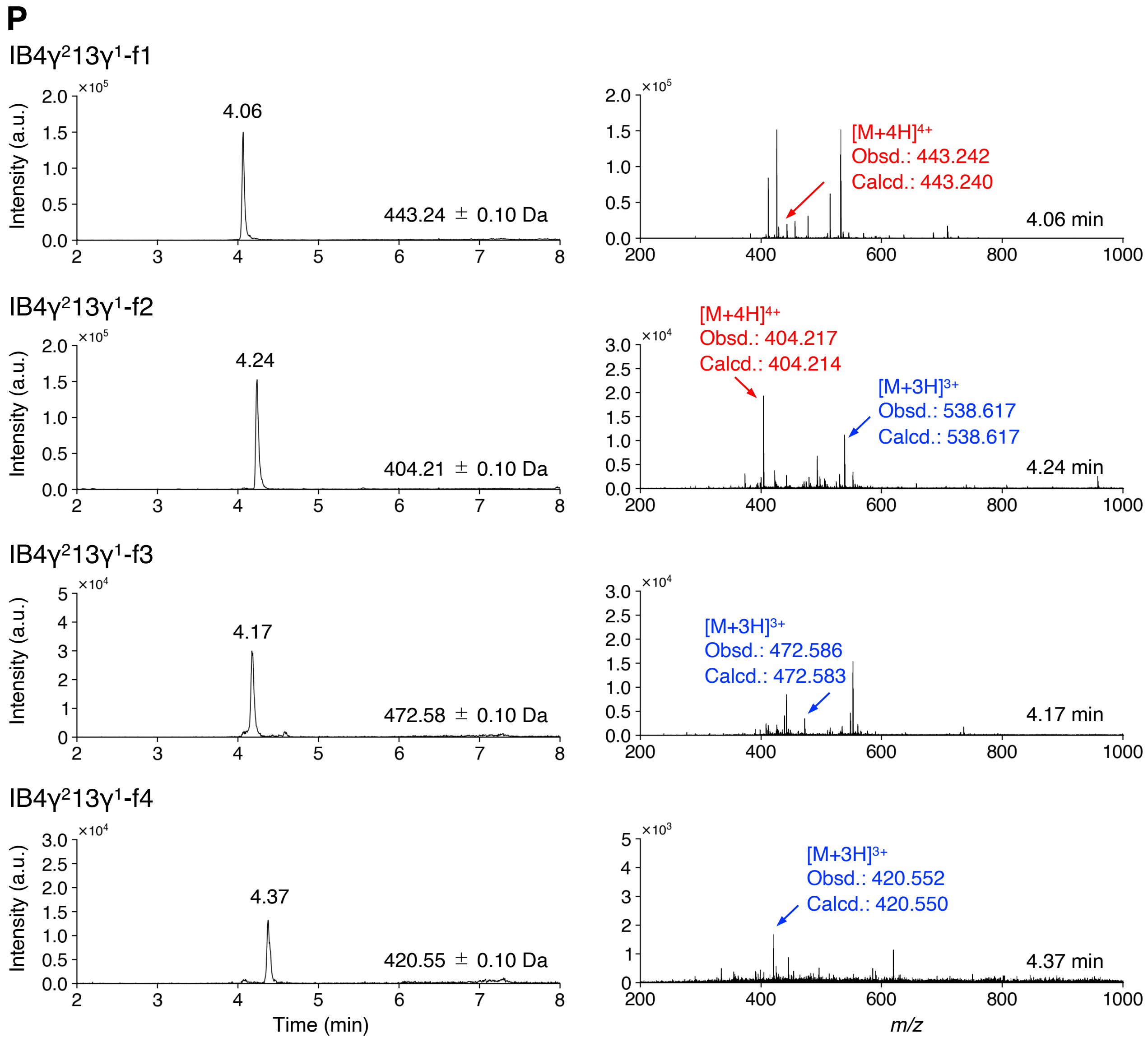

Figure S8, continued.

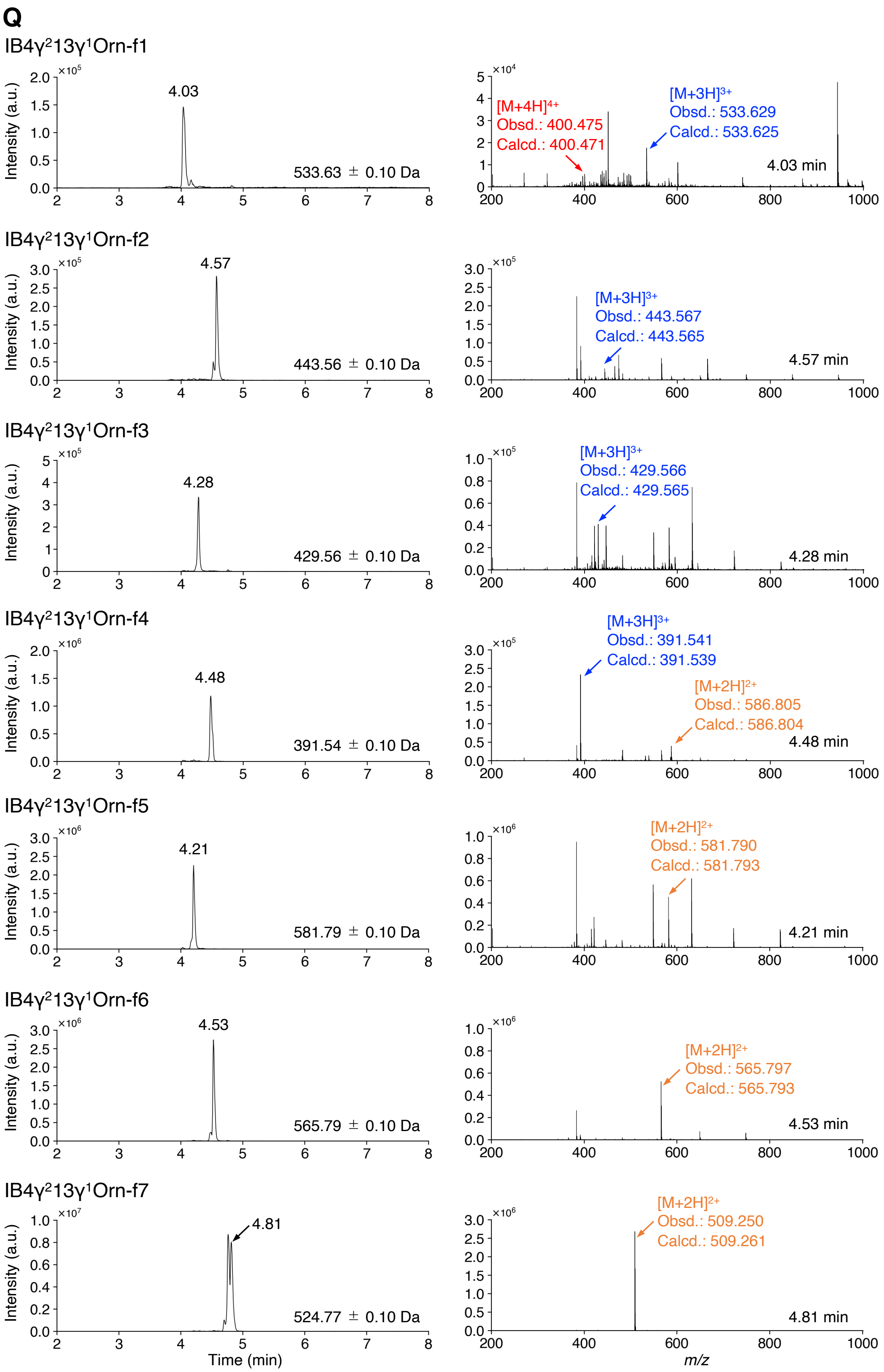

Figure S8, continued.

R

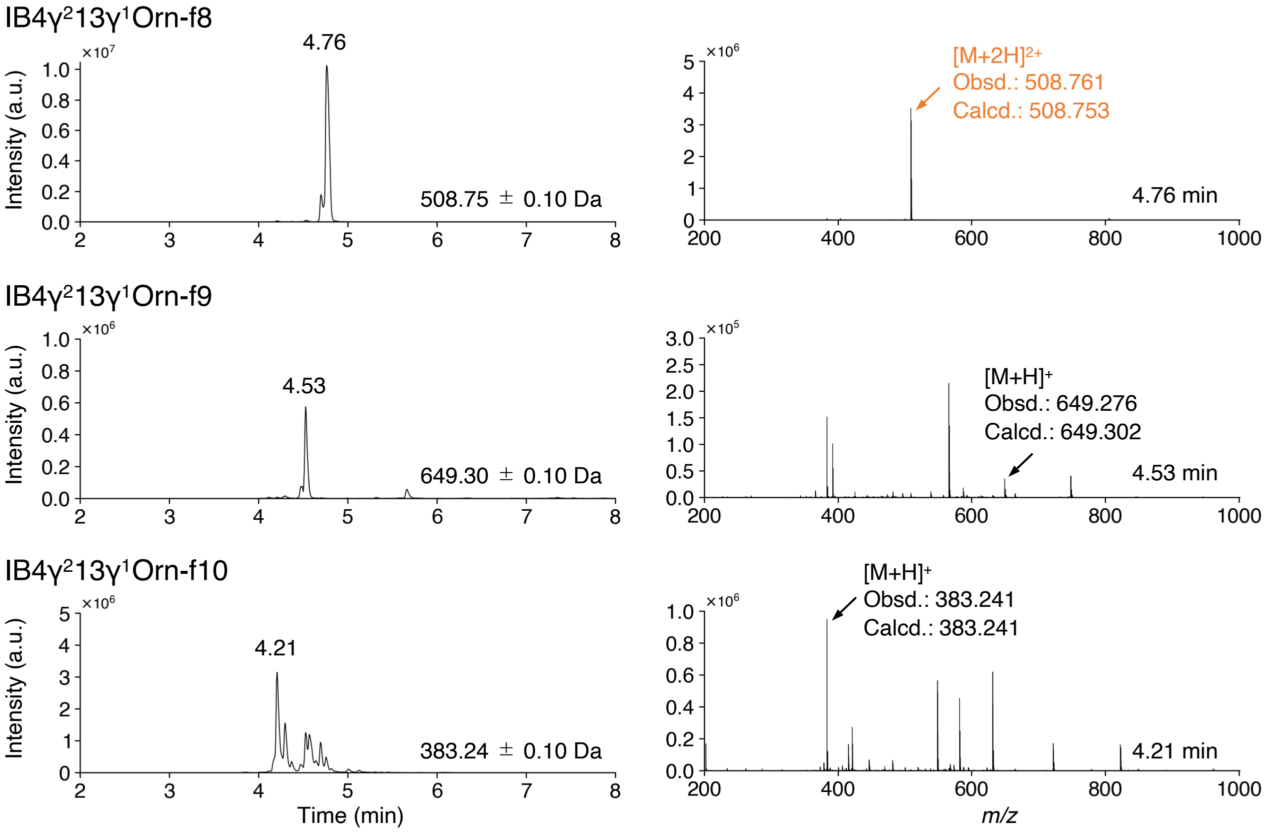

Figure S8, continued.

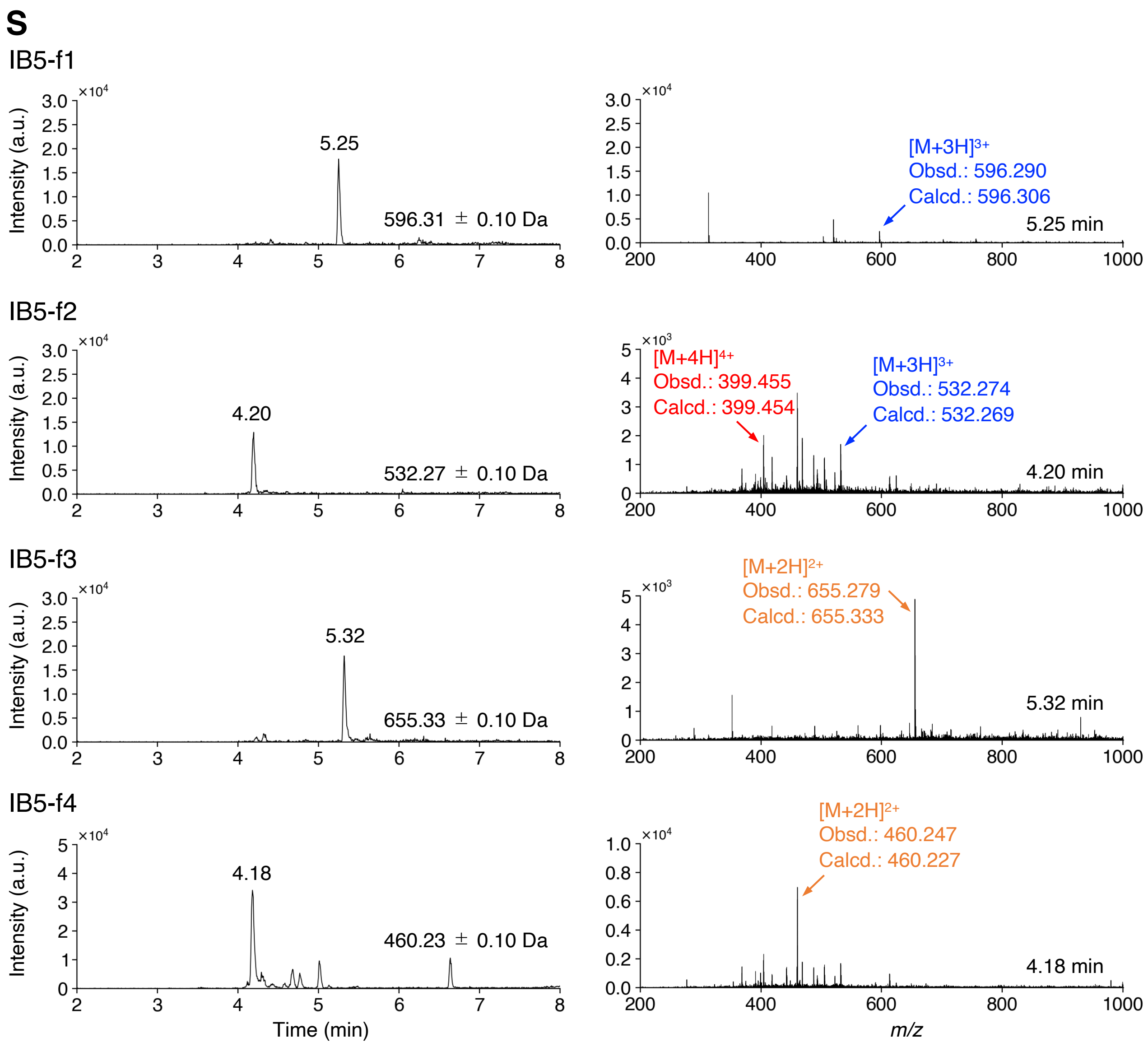

Figure S8, continued.

T

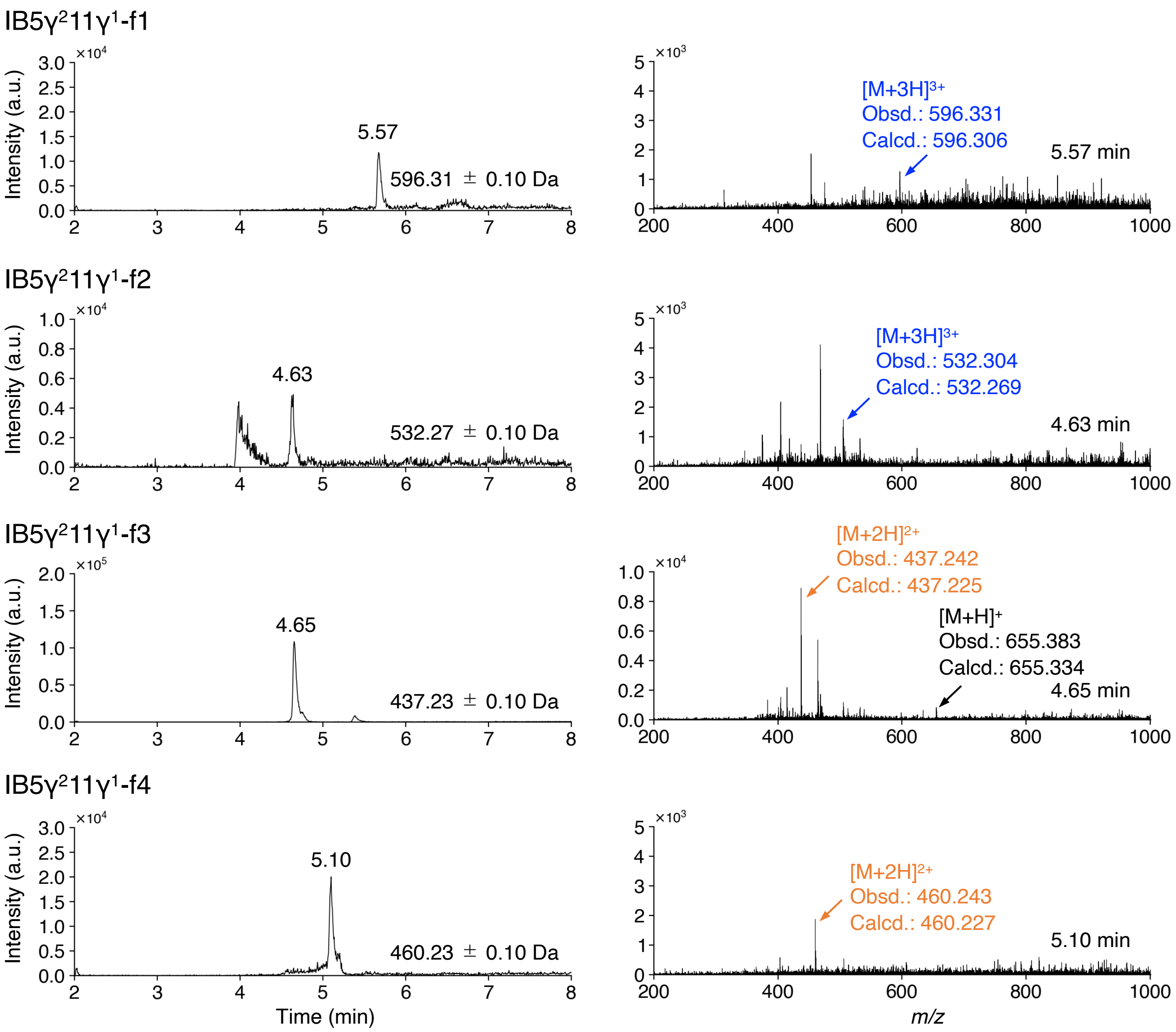

Figure S8, continued.

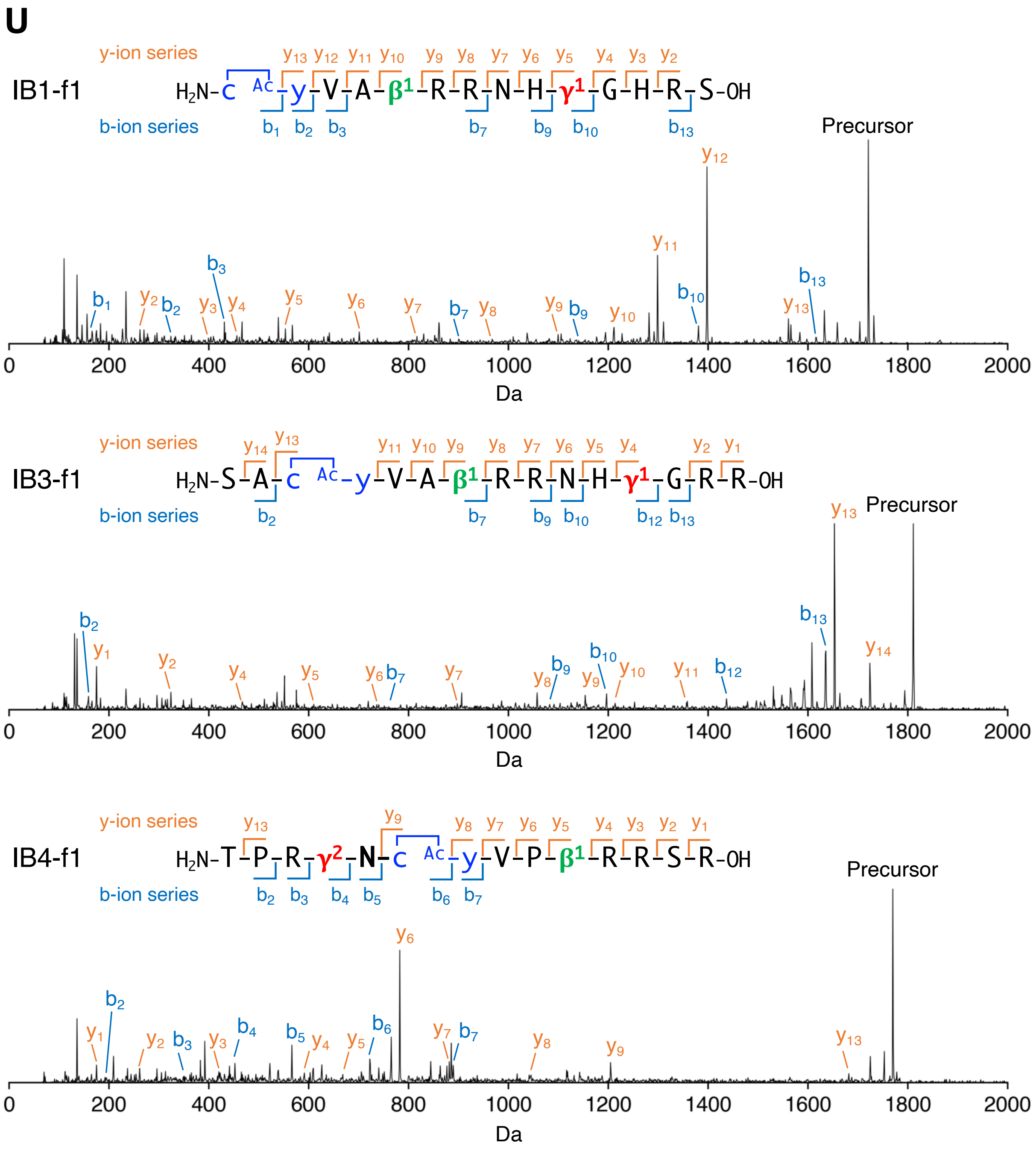

Figure S8, continued.

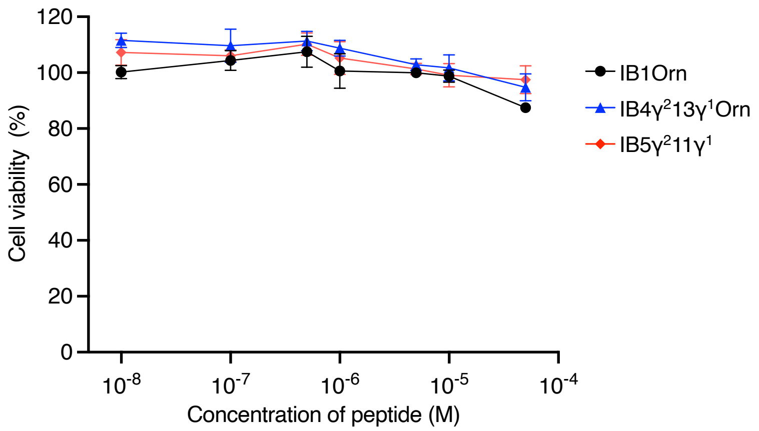

Figure S9. Cell viability assay of IB1Orn, IB4γ^{2}13γ^{1}Orn, and IB5γ^{2}11γ^1 on HEK-Dual^{TM} cells. Cell viability was determined using the Cell Counting Kit-8 (CCK-8) after a 24 h incubation with the peptide. Data are presented as mean ± SD (*n* = 3).

Materials and methods

Materials

Amino acids and their derivatives were purchased from Watanabe Chemical Industries, Enamine, SantaCruz, or BLD Pharmatech. Other chemicals were sourced from Nacalai Tesque, Fujifilm Wako Chemicals, Sigma Aldrich, or Tokyo Chemical Industry. All chemical reagents obtained from commercial sources were used without purification. Recombinant Fc-tagged human IFNGR1 was purchased from ACROBiosystems. Oligonucleotides were procured from Eurofins Genomics (OPC purification grade).

Preparation of flexizymes and tRNAs

Flexizymes (dFx and eFx) and tRNAs were transcribed in vitro using the T7 RNA polymerase from the corresponding template DNAs and prepared by extension and PCR (See SI Table S2 for primer sequences). Extension and PCR was carried out using the following reaction mixture: 50 mM KCl, 10 mM Tris-HCl (pH 9.0), 0.1% (v/v) Triton X-100, 2.5 mM $MgCl_2$ 250 µM dNTP mix, and 60 nM *Taq* DNA polymerase. 2 µM each of forward and reverse extension primers were added to the extension mixture. Extension reaction was initiated at 95 °C for 1 min followed by 5 cycles of 50 °C for 1 min and 72 °C for 1 min. The resulting solution and 0.5 µM each of forward and reverse PCR primers were added to the PCR mixture. PCR was performed for 15 cycles of 95 °C for 40 s, 55 °C for 40 sec, and 72 °C for 40 sec. PCR product was purified by phenol/chloroform extraction and ethanol precipitation. Transcription of RNA was carried out at 37 °C for 16 h using the following mixture: 40 mM Tris-HCl (pH 8.0), 22.5 mM $MgCl_2$, 10 mM dithiothreitol, 1 mM spermidine, 0.01% Triton X-100, 3.75 mM (tRNAs) or 5 mM (flexizymes) NTP mix, 0.04 U/µL RNasin RNase inhibitor (Promega, N2615), and 120 nM T7 RNA polymerase. For transcription of tRNAs, 5 mM GMP was added to the above solution to introduce a monophosphate at the 5′ end of tRNAs. The resulting RNA transcripts were then treated with RQ1 DNase (Promega, M6101) at 37 °C for 30 min and purified by 12% (flexizymes) or 8% (tRNAs) denaturing polyacrylamide gel electrophoresis (PAGE) containing 6 M urea.

Preparation of aminoacyl-tRNAs

D-Cysteine (c), cβAAs, and cγAAs were pre-activated as their 3,5-dinitrobenzyl esters; *N*-chloroacetyl-D-tyrosine (^{ClAc}y) was activated as its cyanomethyl ester.[2,3,4] These activated amino acids were charged onto the respective tRNAs using flexizymes (dFx for 3,5-dinitrobenzyl ester or eFx for cyanomethyl ester). Aminoacylation was carried out at 4 °C for 6 h for c, for 16 h for cβAAs and cγAAs, or 2 h for ^{ClAc}y in the following mixture: 600 mM $MgCl_2$, 20% DMSO, 25 µM dFx or eFx, 25 µM tRNA, and 5 mM activated amino acid. The reaction pH was adjusted by bicine-KOH (pH 8.7) for cβAAs and cγAAs, or HEPES-KOH (pH 7.5) for c and ^{ClAc}y. The reaction was stopped by addition of 4× volume of 0.3 M sodium acetate (pH 5.2) and 10× volume of ethanol. The resulting aminoacyl-tRNAs were washed with ethanol precipitation.

RaPID selection of peptides against IFNGR1

The random mRNA library was ligated with a puromycin linker at the 3′ end, and added to the following translation mixture: 50 mM HEPES-KOH (pH 7.6), 100 mM KOAc, 12.3 mM $Mg(OAc)_2$, 2 mM ATP, 2 mM GTP, 1 mM CTP, 1 mM UTP, 20 mM creatine phosphate, 2 mM spermidine, 1 mM dithiothreitol, 1.5 mg/mL *E. coli* total tRNA, 1.2 µM *E. coli* ribosome, 2.7 µM initiation factor 1 (IF1), 3 µM IF2, 1.5 µM IF3, 20 µM elongation factor Tu/Ts (EF-Tu/Ts), 5 µM EF-P, 0.1 µM EF-G, 0.25 µM release factor 2 (RF2), 0.17 µM RF3, 0.5 µM ribosome recycling factor (RRF), 4 µg/mL creatine kinase, 0.1 µM T7 RNA polymerase, 3 µg/mL myokinase, 0.1 µM inorganic pyrophosphatase, 0.1 µM nucleotide diphosphate kinase, L-α-amino acids (500 µM each A/D/G/H/K/P/T/Y, 250 µM each R/S/V and 125 µM N), 0.73 µM AlaRS, 0.03 µM ArgRS, 0.38 µM AsnRS, 0.13 µM AspRS, 0.09 µM GlyRS, 0.02 µM HisRS, 0.11 µM LysRS, 0.16 µM ProRS, 0.04 µM SerRS, 0.09 µM ThrRS, 0.02 µM TyrRS, 0.02 µM ValRS, 20 µM c-$tRNA^{Pro1E2}_{CAU}$, 20 µM β^1-$tRNA^{Pro1E2}_{GAU}$, 20 µM β^2-$tRNA^{GluE2}_{GCA}$, 50 µM γ^1-$tRNA^{Pro1E2}_{GAA}$, 50 µM γ^2-$tRNA^{Pro1E2}_{GAG}$, and 20 µM ^{ClAc}y-$tRNA^{fMet}_{CAU}$ (SI Figure S3A, step 1). The peptide library was translated at 37 °C for 40 min in 150 µL (for the first round of selection) or 10 µL (from the second round) of the FIT system and incubated at 25 °C for 5 min to conjugate the translated peptide with the corresponding mRNA–puromycin (step 2). 0.04× volume of 500 mM EDTA (pH 8.0) was then added and incubated at 37 °C for 10 min to dissociate ribosomes from the mRNA–peptide conjugates. Reverse transcription (42 °C, 30 min) used the PCR reverse primer (see SI Table S2 for the sequence) and M-MLV reverse transcriptase lacking RNase H activity (Promega, M3682; step 3). The resulting cDNA/mRNA/peptide conjugates were subjected to naked Dynabeads Protein G (Thermo Fisher, DB10004) treatment (4 °C, 15 min) three times to remove bead-binding peptides; the supernatant was then applied to IFNGR1-immobilized Dynabeads (4 °C, 15 min; step 4). The beads were washed with 100 µL ice-cold TBS-T buffer (50 mM Tris-HCl (pH 7.6), 150 mM NaCl, 0.05% (v/v) Tween 20) three times. Note that the removal of bead-binding peptides was not performed for the first selection round. Then, 100 µL of ×1 PCR buffer (10 mM Tris-HCl (pH 9.0), 50 mM KCl, 0.1% (v/v) Triton X-100, 0.25 mM dNTP, 2.5 mM $MgCl_2$, 0.25 µM each PCR forward and reverse primers (see SI Table S2 for the sequence)) was added to the beads; the cDNAs were eluted at 95 °C for 5 min and PCR amplified to make a cDNA library (step 5). To estimate the recovery rate of cDNA, 1 µL of the elute was mixed with 19 µL of 1× PCR buffer containing SYBR Green I (Lonza, 50513) and Taq DNA polymerase; amounts of cDNA were quantified by real-time PCR.

Solid-phase peptide synthesis

Macrocyclic peptides were synthesized by standard Fmoc solid-phase peptide synthesis using a Syro I automated peptide synthesizer (Biotage). NovaPEG Rink Amide Resin (54 mg, 25 µmol) was incubated with *N*,*N*-dimethylformamide (DMF) at room temperature for 1 h. Each Fmoc-protected amino acid was coupled at 30 °C for 40 min on the resin in a DMF solution containing 0.2 M Fmoc-protected amino acid (6 equiv.), 0.2 M 2-(1H-benzotriazole-1-yl)-1,1,3,3-tetramethyluronium

hexafluorophosphate (HBTU; 5 equiv.), 0.2 M 1-hydroxybenzotriazole (HOBt; 5 equiv.) and 0.1 M *N*,*N*-diisopropylethylamine (DIPEA; 12 equiv.). After washing the resin five times with 600 μL DMF, the Fmoc group was deprotected with 600 μL of 40% (v/v) piperidine in DMF at 30 °C for 12 min. Coupling of the Fmoc-protected amino acid and Fmoc deprotection were repeated accordingly. After automated peptide synthesis, 0.2 M chloroacetyl *N*-hydroxysuccinimide ester (8 equiv.) in *N*-methylpyrrolidone was added to the resin; the mixture was incubated at room temperature for 1 h with rotation. After washing the resin subsequently with DMF five times and with dichloromethane five times, the resin-bound peptides were treated with 2 mL of a solution of 92.5% (v/v) TFA, 2.5% (v/v) water, 2.5% triisopropylsilane, and 2.5% 3,6-dioxa-1,8-octanedithiol at room temperature for 3 h with rotation to deprotect protecting groups and cleave off from the resin. The resulting linear peptides were precipitated with diethyl ether and dissolved in 10 mL of 80% (v/v) DMSO and 0.1% TFA in water. Following the addition of 200 μL of 0.5 M tris(2-carboxyethyl) phosphine (TCEP) and triethylamine to adjust the pH to 8, the peptide mixture was incubated with rotation at room temperature for 16 h to form a thioether bond between the N-terminal chloroacetamide and thiol group of the downstream cysteine. To prevent undesired intermolecular reactions, the concentration of peptides was kept below 1 mM during the intramolecular macrocyclization reaction. The resulting macrocyclic peptides were purified by reverse-phase HPLC (Shimadzu) with Chromolith Prep RP-18 column (Merck). Macrocyclization of the peptides was confirmed by MALDI-TOF MS, where no byproducts derived from the intermolecular reactions were detected.

Evaluation of binding affinity of peptides by surface plasmon resonance

The binding affinities of selected peptides and IFNGR1 were analyzed by surface plasmon resonance (SPR) using a Biacore T200 instrument (Cytiva) at 25 °C with the following running buffer: 10 mM Tris-HCl (pH 8.0), 150 mM NaCl, 0.05% (v/v) Tween 20, and 0.1% (v/v) DMSO. Fc-tagged IFNGR1 was immobilized on a series S sensor chip protein G (Cytiva) to a surface density of 1,200–1,600 response units following the immobilization protocols provided by Cytiva. The kinetic constants were determined by a single-cycle kinetics method by the injection of five different concentrations (twofold dilution series) of each peptide at a flow rate of 30 μL/min. The resulting sensorgrams were fitted to the standard 1:1 interaction model and analyzed using Biacore evaluation software.

IFNGR1 inhibition assay using AlphaLISA

The inhibitory activity of peptides against IFN-γ/IFNGR1 protein–protein interaction (PPI) was determined by AlphaLISA.[5] Peptides, proteins, and beads were diluted in AlphaLISA immunoassay buffer (PerkinElmer, AL000C). 3.6 μL of 5 nM Fc-tagged IFNGR1 and 3.6 μL of synthetic peptides were mixed in AlphaPlate-384 Shallow Well (PerkinElmer) and preincubated at 25 °C for 10 min. 3.6 μL of 15 nM biotin-tagged IFN-γ was then added to the mix and incubated at 25 °C for 20 min. 3.6 μL each human IgG acceptor beads (PerkinElmer) and streptavidin donor beads (PerkinElmer) were added to the above mix and incubated at 25 °C for 30 min in dark place. Emission at 615 nm was measured

by EnSpire Multimode Plate Reader (PerkinElmer). The fraction of bound IFN-γ/IFNGR1 was calculated by the intensity of emission in the presence of peptide divided by that in the absence of peptide. IC_{50} value was analyzed by a four-parameter dose-response curve fitting by using GraphPad Prism 9.

Serum stability assays

10–200 μM synthetic peptide and 5 μM serum-resistant internal standard peptide consisting only of D-amino acids (NH_2-PEG_5-wstndwstnd-PEG_5-$CONH_2$)[3] were mixed and incubated in human serum (Cosmo Bio, 12181201) at 37 °C for up to 120 h. At each time point, 4 μL of the mixture was removed and quenched by adding 12 μl methanol. Following centrifugation (15,000 g, 4 °C, 5 min), 10 μL of the supernatant was mixed with 40 μl of 1% (v/v) TFA. Following centrifugation (15,000 g, 4 °C, 5 min), the supernatant was collected for LC/MS analysis, which used a reverse-phase column (ACQUITY UPLC BEH C18, 1.7 μm, 2.1 × 150 mm; Waters) and a Xevo G2-XS QTof system (Waters) with a linear gradient from 1% B to 61% B. Buffer A was water with 0.1% (v/v) formic acid; buffer B was acetonitrile with 0.1% (v/v) formic acid. The percentages of remaining peptides were determined by the peak area integration of the chromatograms. For tandem mass spectrometry, MS/MS spectra were acquired with a 0.2 s scan time, with 10−30 eV collision energy. The obtained LC/MS data was analyzed using MassLynx 4.1 (Waters).

HEK-Dual™ IFN-γ/IFNGR reporter assay

HEK-Dual™ IFN-γ cells (InvivoGen) were cultured following the manufacturer's instructions in DMEM supplemented with 10% FBS, 4.5 g/mL glucose, 2 mM L-glutamine, 100 μg/mL penicillin-streptomycin, 100 μg/mL Normocin, 30 μg/mL Blasticidin, 100 μg/mL Zeocin, and 1 μg/mL Puromycin. Normocin, Blasticidin, Zeocin, and Puromycin were excluded when conducting the experiments. To evaluate the cellular activity of peptides, peptide mixtures were added to 50,000 cells/well in a flat-bottom 96-well plate and pre-incubated for 30 min at 37 °C before stimulation. Subsequently, 1 ng/mL of recombinant IFN-γ (InvivoGen) was added to each well in a final reaction volume of 200 μL, followed by incubation for 20 h at 37 °C. 20 μL of supernatants from the induced cells was added to 180 μL QUANTI-Blue™ solution (InvivoGen). After further incubation for 3 h at 37 °C, the expression levels of the secreted embryonic alkaline phosphatase (SEAP) were determined by reading the O.D. at 650 nm on a Tecan M1000 PRO microplate reader (Tecan Group) and normalized to the non-treated well. IC_{50} value was analyzed by a normalized dose-response curve fitting by using GraphPad Prism 9.

Cell viability assay

HEK-Dual™ IFN-γ cells were used for the cell viability assay. 10,000 cells/well were added to each well in a flat-bottom 96-well plate. After 24 h, peptide mixtures were added to each well in a final volume of 100 μL. After further incubation for 24 h, 10 μL CCK-8 reagent (Dojindo) was added to

each well and incubated for 3 h at 37 °C. Live cell counts were determined by reading the O.D. at 450 nm on a Tecan M1000 PRO microplate reader (Tecan Group) and normalized to the non-treated well.

References for Supporting Information

(1) Miura, T.; Malla, T. R.; Owen, C. D.; Tumber, A.; Brewitz, L.; McDonough, M. A.; Salah, E.; Terasaka, N.; Katoh, T.; Lukacik, P.; Strain-Damerell, C.; Mikolajek, H.; Walsh, M. A.; Kawamura, A.; Schofield, C. J.; Suga, H., In vitro selection of macrocyclic peptide inhibitors containing cyclic $\gamma^{2,4}$-amino acids targeting the SARS-CoV-2 main protease. *Nat. Chem.* 2023, *15* (7), 998-1005.

(2) Goto, Y.; Katoh, T.; Suga, H., Flexizymes for genetic code reprogramming. *Nat. Protoc.* **2011**, *6* (6), 779-790.

(3) Katoh, T.; Sengoku, T.; Hirata, K.; Ogata, K.; Suga, H., Ribosomal synthesis and de novo discovery of bioactive foldamer peptides containing cyclic β-amino acids. *Nat. Chem.* **2020**, *12* (11), 1081-1088.

(4) Katoh, T.; Suga, H., Ribosomal elongation of cyclic γ-amino acids using a reprogrammed genetic code. *J. Am. Chem. Soc.* **2020**, *142* (11), 4965-4969.

(5) Katoh, T.; Suga, H., In vitro selection of foldamer-like macrocyclic peptides containing 2-aminobenzoic acid and 3-aminothiophene-2-carboxylic acid. *J. Am. Chem. Soc.* **2022**, 144 (5), 2069-2072.
